# A ligand-based computational drug repurposing pipeline using KNIME and Programmatic Data Access: case studies for rare diseases and COVID-19

**DOI:** 10.1186/s13321-020-00474-z

**Published:** 2020-11-25

**Authors:** Alzbeta Tuerkova, Barbara Zdrazil

**Affiliations:** Department of Pharmaceutical Chemistry, Division of Drug Design and Medicinal Chemistry, University of Vienna, Althanstraße 14, 1090 Vienna, Austria

**Keywords:** Drug repurposing, Data integration, Data mining, Data access, Application programming interface, Substructure search, Rare disease, KNIME workflow, COVID-19, SARS-CoV-2, GLUT-1 deficiency syndrome, ChEMBL, Open targets platform, DrugBank, PDB, UniProtKB, Guide-to-pharmacology, PubChem

## Abstract

Biomedical information mining is increasingly recognized as a promising technique to accelerate drug discovery and development. Especially, integrative approaches which mine data from several (open) data sources have become more attractive with the increasing possibilities to programmatically access data through Application Programming Interfaces (APIs). The use of open data in conjunction with free, platform-independent analytic tools provides the additional advantage of flexibility, re-usability, and transparency. Here, we present a strategy for performing ligand-based in silico drug repurposing with the analytics platform KNIME. We demonstrate the usefulness of the developed workflow on the basis of two different use cases: a rare disease (here: Glucose Transporter Type 1 (GLUT-1) deficiency), and a new disease (here: COVID 19). The workflow includes a targeted download of data through web services, data curation, detection of enriched structural patterns, as well as substructure searches in DrugBank and a recently deposited data set of antiviral drugs provided by Chemical Abstracts Service. Developed workflows, tutorials with detailed step-by-step instructions, and the information gained by the analysis of data for GLUT-1 deficiency syndrome and COVID-19 are made freely available to the scientific community. The provided framework can be reused by researchers for other in silico drug repurposing projects, and it should serve as a valuable teaching resource for conveying integrative data mining strategies.

## Background

Computer-aided mining of biomedical data is an emerging field in cheminformatics and drug design which has reshaped current drug development [1–3]. Open access to various life-science repositories, such as ChEMBL [4], PubChem [5], UniProt [6], or DrugBank [7], provides a competitive advantage when using data-driven drug discovery approaches as opposed to non-integrative approaches [8]. Furthermore, many databases enable programmatic access of the stored data through an Application Programming Interface (API). Consequently, it is of importance to find appropriate tools to analyze gathered data in an automated way. The Konstanz Integration Miner (KNIME) is an open-source data pipelining and analytics platform which enables the creation of (semi)automated workflows to process, transform, analyze, and visualize the data as well as the generation and deployment of approximative mathematical models [9]. In the recent past, the KNIME community has released a plethora of cheminformatics extensions, such as the RDKit [10], Chemistry Development Kit (CDK) [11], Indigo [12], or Vernalis [13] toolkits.

Large-scale data fusion supplied with cheminformatics data analyses can uncover underlying patterns within the data and can pave the way for the development of novel medicine. Such a strategy can be leveraged for drug repurposing (also known as drug repositioning) strategies, in which a re-evaluation of an already approved drug can lead to a treatment for another disease [14]. This approach is particularly useful to, e.g., discover a cure for orphan diseases [15], or to find drug candidates that are worth further investigations for an ongoing pandemic, such as Coronavirus disease 2019 (COVID-19).

With the rapid increase of the availability of biomedical data in the open domain, computational drug repurposing approaches now strongly benefit from interconnecting different types of data entities, including genes, tissue expression data, targets, drugs, phenotypes, and diseases, to deliver an indication about a drugs’ mode-of-action. Method-wise, computational drug repurposing techniques range from data (text) mining, and different machine learning approaches, to network analyses and structure-based approaches [16]. For example, Li et al. combined data originating from text mining with protein interaction networks to develop a drug-target connectivity map for a certain disease [17]. Machine learning methods used in drug repurposing strategies include, *inter alia*, support vector machines [18], classification models [19], and currently also deep neural networks [20] to predict drug-disease relationships. Network analyses enable to model complex functional similarities between various biological entities, such as drugs, genes, proteins, or entire protein families [21]. An orthogonal approach to ligand-based strategies, is to perform structure-based virtual screening by using a consensus inverse docking strategy, as demonstrated by Wang et al. [22].

Semi-automated drug repositioning pipelines are uniting the advantages of computational workflows (e.g., provided by using the open source tool KNIME) with the availability of big open data sources that can be accessed programmatically. They make access to data resources easier and thus lower the barriers for effective data usage for non-data scientists. Also, their usage shortens the time period from data collection to the identification of hidden relationships in the data. In addition, such workflows are easily reproducible, and can be adapted according to individual project needs [23].

In this study, we are providing a general strategy and a step-by-step tutorial for automated data access and data integration from multiple open data sources (which are providing an API), along with data processing and cheminformatics data analysis by using the pipelining tool KNIME. Individual operations, such as the specification and execution of API requests, extraction of properties through JSON/XPath queries, structural data standardization, identification of enriched structural fragments, and substructure searches in external data sources, are thoroughly described and demonstrated herein.

Protein and ligand information related to GLUT-1 deficiency syndrome and to COVID-19 have been chosen as individual use cases to demonstrate the usefulness of the approach. GLUT-1 deficiency syndrome is a rare disease caused by genetic variation of glucose transporter member 1 (SLC2A1), which leads to impaired transport of glucose (https://ghr.nlm.nih.gov/condition/glut1-deficiency-syndrome). For COVID-19, to date only data for suggested targets can be used (with relatively little knowledge about the strength of the target-disease associations). Just recently, about 66 druggable protein targets with potential interest for SARS-CoV-2 treatment have been reported [24].

The Open Targets Platform integrates public domain data to enable target identification and prioritization by providing association scores between targets and diseases [25]. Targets represented in the Open Targets Platform can be genes, transcripts or proteins integrated through the Ensembl gene ID (https://www.ensembl.org/index.html).

In this study, we used highly scored proteins from the Open Targets Platform for the diseases under investigation. In case of COVID-19, protein targets listed in the UniProtKB pre-release web page (available at https://covid-19.uniprot.org/uniprotkb?query=* ) were additionally used as a starting point.

**Fig. 1:**
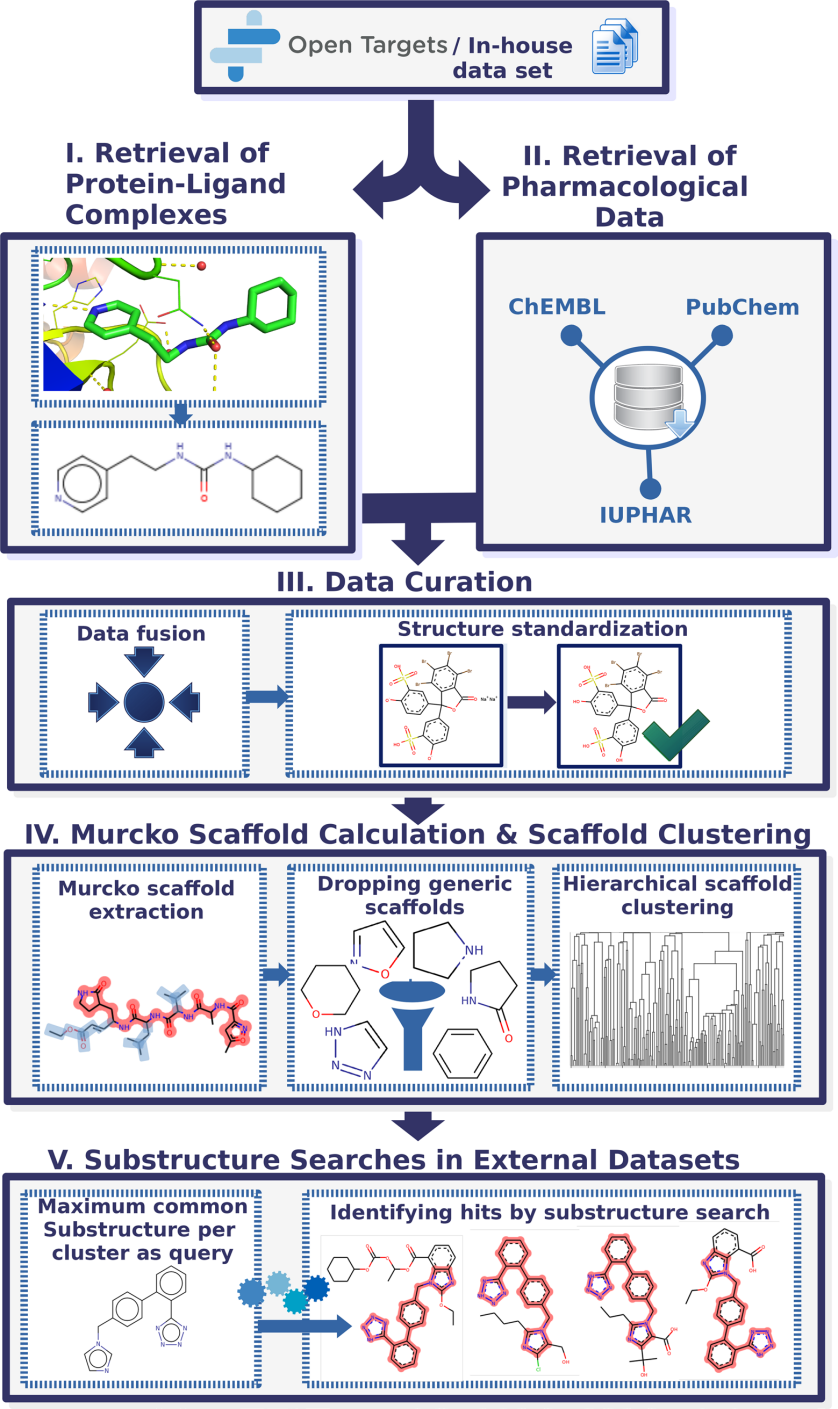
Schematic overview of the data-driven drug-repurposing workflow

API calls were specified to map UniProt IDs of the targets to available structural data in the Protein Data Bank (PDB) [26]. Ligands co-resolved with a protein structure were extracted as separate entities. For sake of data augmentation, ligand bioactivity measurements (such as Ki, IC50, or Km end-points) for the protein targets under study were retrieved from ChEMBL [4], PubChem [5], and Guide-to-Pharmacology (IUPHAR) [27]. After data cleaning and chemical structure standardization, Bemis-Murcko scaffolds [28] were extracted from the ligands in the data set and grouped by similarity into structural queries for subsequent substructure searches in DrugBank [7] and the CAS COVID-19 antiviral candidate compounds data set (available upon request at https://www.cas.org/covid-19-antiviral-compounds-dataset). These searches led to the identification of structurally analogous compounds which could potentially show similar pharmacological action at targets associated with GLUT-1 deficiency syndrome or COVID-19. A list of identified hits, is provided as an output of the workflow. A schematic overview of the whole data-driven drug repurposing workflow is depicted in Fig. 1.

Taken together, the developed data mining pipeline is a useful resource for any in silico drug repurposing project and is exemplified on the basis of a drug repositioning strategy for GLUT-1 deficiency syndrome and the Coronavirus Disease 2019 (COVID-19). The step-by-step instructions allow for an easy implementation for other drug discovery projects along these lines and they shall give especially guidance to students or researchers new to the field of data-driven drug discovery. All workflows can be accessed via an open GitHub Repository (available at https://github.com/AlzbetaTuerkova/Drug-Repurposing-in-KNIME).

## Methods

As the drug-repurposing strategy applied here is mainly conceived for educational purposes, we introduce a step-by-step tutorial for a guided development of a KNIME workflow. In addition, the workflow developed herein is fully versatile and it can thus be reproduced for other diseases of interest. Basic knowledge of configuration and execution of standard nodes (e.g., the ‘Row Filter’ node, the ‘GroupBy’ node, the ‘Joiner’ node, the ‘Pivoting’ node), import of external data sets into a KNIME workflow (e.g., the ‘SDF reader’ node, the ‘File Reader’ node), handling different structural formats, as well as working with specific data types in KNIME, is expected here as a prerequisite.

When integrating data from diverse sources, it becomes beneficial to query databases programmatically, i.e., without the need of laborious manual data download and data integration. UniProtKB and other databases used in this example enable targeted access of the stored data through an Application Programming Interface (API).

**Fig. 2:**
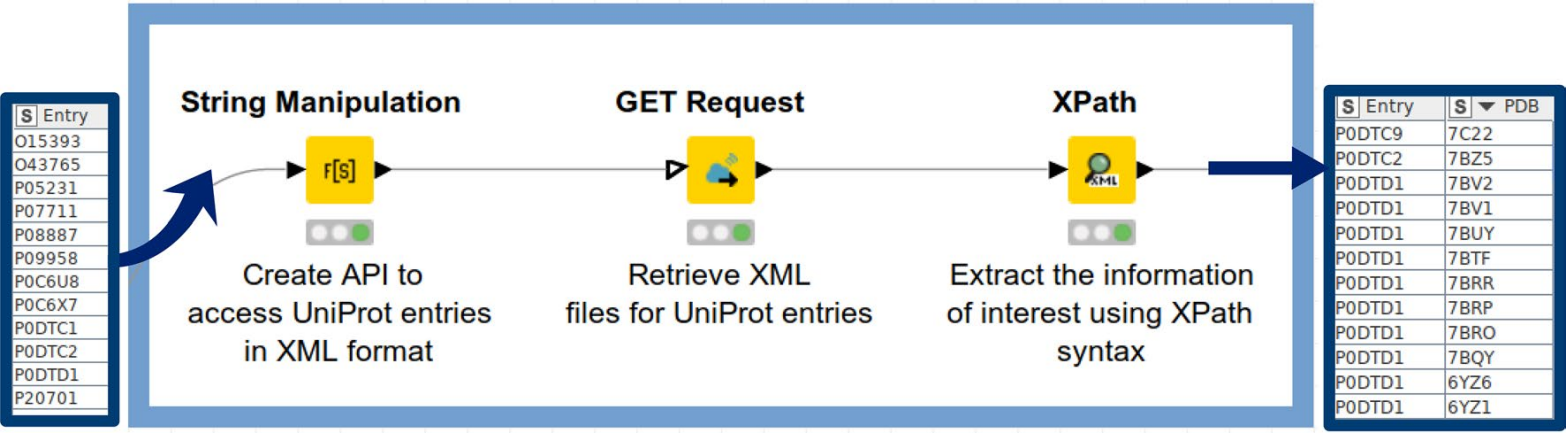
An example workflow for creation, execution, and post-processing API requests to retrieve protein information from UniProt: The ‘String Manipulation’ node accepts a column with UniProt IDs listed and an API request is created (as string). The ‘GET Request’ node executes the requests generated in the previous step. The ‘XPath’ node extracts PDB IDs associated with a certain UniProt ID (if available). For a detailed description of how to retrieve UniProt IDs we refer the reader to the subsequent section

In the KNIME workflow discussed herein, a triad of KNIME nodes is consecutively executed (1) to specify the API request (via the ‘String Manipulation’ node), (2) to retrieve data from web services (via the ‘GET request’ node), and (3) to perform XPath/JSON queries to extract useful properties for a given protein (via the ‘XPath’ or ‘JSONPath’ node, respectively). The corresponding part of the KNIME workflow is depicted in Fig. 2.

### 1. Step: Mapping target identifiers of the Open Targets Platform to UniProt

The workflow discussed herein, allows two different sorts of input: (1) Automated retrieval of targets associated with a certain disease via the Open Targets Platform and (2) importing an external data set with a list of protein targets.

In option (1), the disease identifiers from the Open Targets Platform for GLUT-1 deficiency syndrome (Orphanet_71277) and COVID-19 (MONDO_0100096) have been specified as input in the ‘Table Creator’ node. Next, an API request to fetch disease records was created using the ‘String Manipulation’ node. The join() function in the ‘String Manipulation’ node is used and a corresponding Open Targets Platform disease ID is forwarded to the string as a variable ($disease_id$ column). Additional parameters used in this API request are the maximum number of associated drug targets (‘size’, here set to 10,000), and the association score, which enables to prioritize the drug targets on basis of their available evidence for a disease (‘scorevalue_min’, here set to 0.99): join("https://platform-api.opentargets.io/v3/platform/public/association/filter?disease=",$disease_id$,"&size=10000&scorevalue_min=0.99").

As an output of the ‘String Manipulation’ node, a column with the respective API requests is appended to the output table, such as: https://platform-api.opentargets.io/v3/platform/public/association/filter?disease=EFO_0001360&size=10000&scorevalue_min=0.99.

**Fig. 3:**
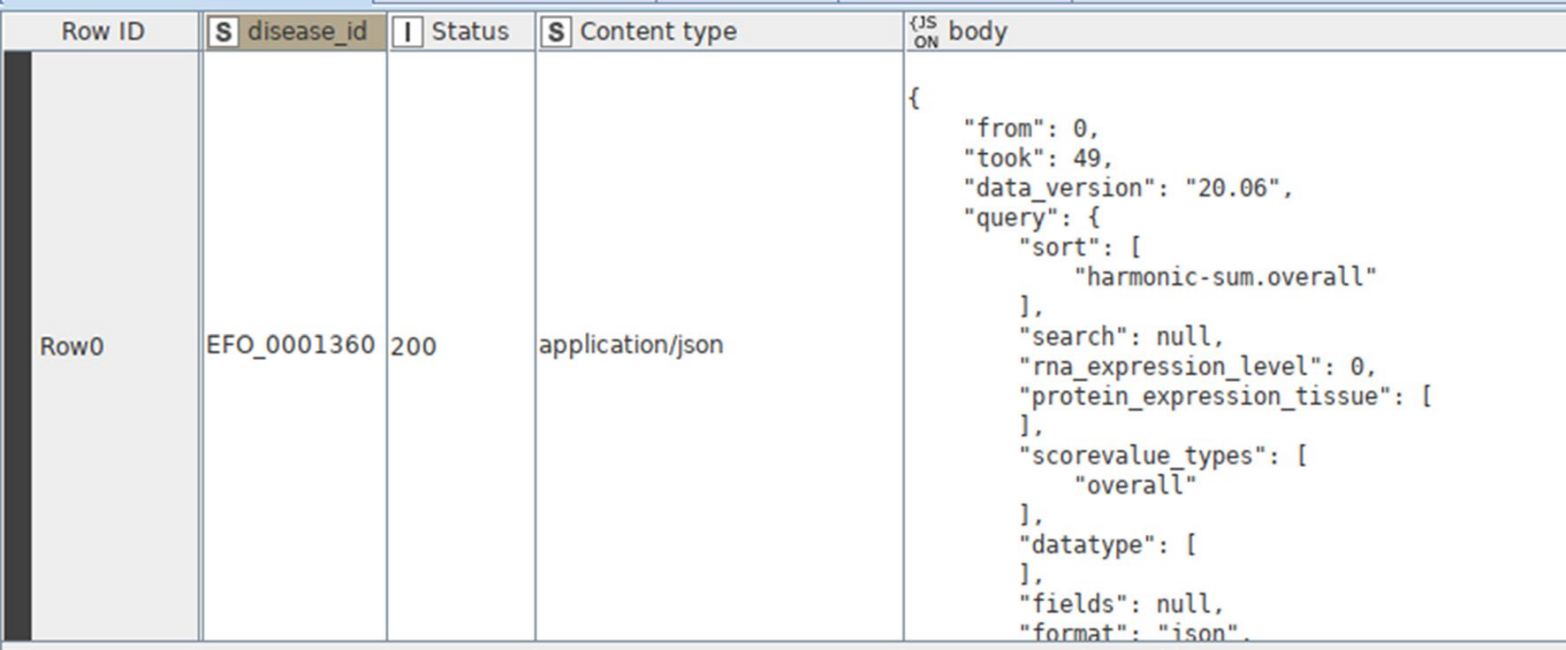
An example of the output table generated after the execution of the ‘GET Request’ node: Status, content type, and JSON file are appended to the table as separate columns

By executing the API request (via the ‘GET Request’ node), a JSON file is downloaded from the Open Targets Platform and appended to the output table as a separate column. Additionally, columns reporting the content type (here ‘application/json’), and the HTTP status code are appended (Fig. 3). There exist five classes of HTTP status codes: (1) Informational responses (100–199), (2) Successful responses (200–299), (3) Redirects (300–399), (4) Client errors (400–499), and (5) Server errors (500–599). The information provided about the status of the request can be used to filter out any useless data entries. It is recommended to increase the timeout in the ‘GET Request’ configuration as the default specification (2 s) is usually insufficient to receive all requested data.

Subsequently, the ‘JSON Path’ node is used to extract the information of interest on the basis of querying different JSON objects. The ‘JSON Path’ node enables to create JSON Path queries in both dot-notation and bracket-notation (depending on how the properties of an object are specified in the syntax). Here, the bracket notation is applied to extract target identifiers, target names, and gene symbol by using the following JSON paths:


$['data'][*]['target']['id']



$['data'][*]['target']['gene_info']['name']



$['data'][*]['target']['gene_info']['symbol']


Output values are appended to separate cells as a collection data type. The ‘Ungroup’ node is subsequently used to transform collections of values into individual rows.

Next, cross-references for all human target entries in the Open Targets Platform can be fetched via the UniProt web services. Here, a corresponding API request was executed to retrieve the mappings for targets (UniProt target IDs are mapped to Open Targets Platform target IDs): https://www.uniprot.org/uniprot/?query=organism:9606+AND+database:OpenTargets&format=xls&columns=id,database(OpenTargets),reviewed.

Due to the potential workflow overload, we recommend to download a mapping file (XLS format) and forward it to the workflow via the ‘File Reader’ node and later join the two data sets via the ‘Joiner’ node.

Option (2) is to use a user-specified list of UniProt IDs in a data table format. In this contribution, this step is exemplified by the use case for proteins that are listed to be of potential interest for treating COVID-19 (53 entries available at https://covid-19.uniprot.org/uniprotkb?query=*). The CSV/TSV file is read in by a ‘File Reader’ node.

### 2. Step: Retrieving protein–ligand structural data from the Protein Data Bank

UniProt IDs for targets of interest were used to retrieve available protein–ligand complexes stored in the Protein Data Bank (PDB) [29].

Based on the same strategy as in step 1, a column with the respective API requests is appended to the output table. An example for such an API request looks like this: https://www.uniprot.org/uniprot/F8W8F0.xml.

When executing the workflow with COVID-19 pre-release data provided by UniProtKB, the API request has to be adopted in the following manner: https://www.ebi.ac.uk/uniprot/api/covid-19/uniprotkb/accession/O15393.xml.

By executing the API requests (via the ‘GET Request’ node), the XML file is downloaded from UniProt and appended to the output table as XML cell. Similar to the ‘JSON Path’ described in the previous step, the ‘Xpath’ node (XPath 1.0 version) is used to extract the information of interest on the basis of querying different XML elements and the associated XML attributes. One can define an Xpath query within the ‘Xpath’ node from scratch. Another way is to perform a double-click on a specific section in the XML-Cell Preview table and the Xpath query is generated automatically. The XPath query below is used to retrieve all available PDB IDs for a given UniProt ID:


/dns:uniprot/dns:entry/dns:dbReference[@type='PDB']/@id


The ‘dns’ prefix corresponds to the namespace used in the XPath query. Here, http://uniprot.org/uniprot’ is used as a namespace. Namespaces are defined automatically and are listed in the node configuration.

The example XPath query shows that PDB IDs are integrated within the < dbReference > XML element. However, UniProt entries consist of multiple < dbReference > elements which are pointing to different data sources, such as PubMed, GO, InterPro, Pfam, or PDB:


<dbReference type="PubMed" id="12730500">



<dbReference type="GO" id="GO:0039579">



<dbReference type="InterPro" id="IPR036333">



<dbReference type="Pfam" id="PF06478">



<dbReference type="PDB" id="6NUR">


A key task is to query data from XML elements which do possess the ‘PDB’ attribute exclusively. The ‘@’ character is used to specify certain XML attributes in the XPath query. Therefore, dbReference[@type = 'PDB'] is forwarded to the XPath query to get all PDB IDs by querying the @id attribute.

Due to the possible synchronization delay of UniProt releases with other cross-referenced databases, an additional alternative approach has been used to fetch PDB data. Specifically, PDBe graph APIs were used for this purpose. The PDB entities are returned in JSON format by default. Below an example is provided for a request to fetch protein structures for the ACE2 receptor (UniProt ID: Q9BYF1) via PDBe graph APIs: https://www.ebi.ac.uk/pdbe/graph-api/mappings/best_structures/Q9BYF1.

Similar to the ‘XPath’ node for processing XML documents, KNIME also provides the ‘JSON Path’ node which is used to process JSON data. The ‘JSON Path’ node enables to create JSON Path queries in both dot notation and bracket notation (depending on how the properties of an object are specified in the syntax). In the discussed KNIME workflow herein, the bracket notation is applied to extract the PDB IDs:


$..[*].['pdb_id']


Since the data are listed as a collection column type, the ‘JSON Path’ node is followed by the ‘UnGroup’ node to list multiple PDB IDs per protein target into separate rows. After concatenating data (‘Concatenate’ node) retrieved from PDBe graph APIs, duplicates for a respective target were removed by grouping the data by target UniProt ID and PDB IDs (‘GroupBy’ node). The ‘PDB ID’ column is used to create the Uniform Resource Locator (URL) path to extract different properties by using the same strategy as shown in Fig. 2. An example of such URL is given below: https://files.rcsb.org/view/2VYI.pdb.

**Fig. 4:**
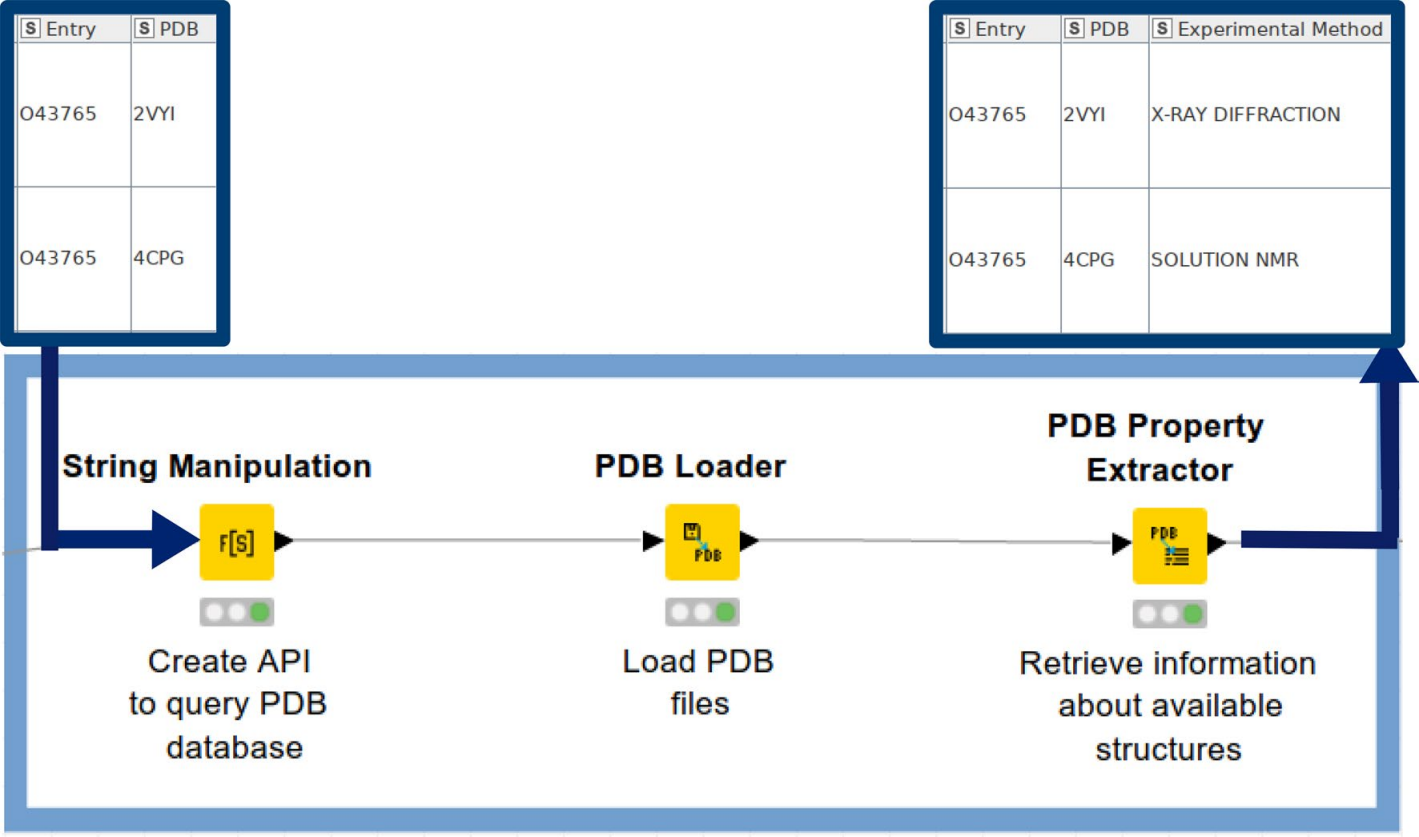
PDB nodes which enable to fetch and extract various properties of deposited PDB structures: The ‘String Manipulation Node’ accepts a column with PDB IDs listed. The ‘PDB Loader’ node is used to download PDB files specified by an URL path. The ‘PDB Property Extractor’ is used to extract structure title, experimental method, crystal resolution (if applicable), number of models (if applicable), R and Rfree factor (if applicable), space group (if applicable), and further experimental remarks from a PDB cell column. An example output table shows UniProt ID (‘Entry’ column), associated PDB IDs (‘PDB’ column), and experimental method (‘Experimental Method’) used for resolving 3D structures

The ‘PDB Loader’ and the ‘PDB Property Extractor’ nodes are available from the KNIME repository (created by Vernalis, Cambridge, UK) to facilitate analysis of PDB data in KNIME (Fig. 4). These nodes were employed in order to explore properties of the PDB files, such as the experimental method used (X-ray diffraction, solution NMR, cryo-EM, theoretical models), the number of stored models, the resolution of structures, Space groups, R-factor, and so on.

Next, the available PDB structures were examined for their availability of co-resolved ligands. Ligand information (in JSON format) can be received through the RCSB PDB RESTful Web services by creating the following request: https://data.rcsb.org/rest/v1/core/entry/2VYI.

Following JSON Path node is used to retrieve a collection of bound ligands, if available:


$['rcsb_entry_info']['nonpolymer_bound_components']


Available ligands are listed using their shortcuts (e.g., BME, NAG, XU3). An API request is subsequently created and executed to fetch ligand information (in JSON format): https://data.rcsb.org/rest/v1/core/chemcomp/NAG.

The following JSON path node is used to retrieve the SMILES code for a specific ligand:


$['rcsb_chem_comp_descriptor']['smiles']


Subsequently, PDB entries without a co-resolved ligand are filtered out (by applying the ‘RowFilter’ node). The ‘GroupBy’ node is used to keep unique ligand structures per protein target (grouping by UniProt ID and smiles string). This procedure might also retrieve salts, solvents, and/or co-crystallizing compounds, as they are identified as ‘ligands’ in PDB. Although the salts and unconnected fragments are stripped during the structure standardization procedure (as described in Sect. 3), it is generally advisable to cross-check the output table to eliminate retained co-crystallizing agents (e.g., isonicotinamide).

### 3. Step: Fetching ligand bioactivity data from open bioactivity data sources via programmatic data access

Orthogonal to fetching ligand data for drug targets of interest from their protein structures, ligands and their experimental bioactivity measurements can also be collected from open pharmacological databases. In this example, data is retrieved from ChEMBL (version 26) [4, 30], PubChem [5], and IUPHAR (also known as Guide-to-Pharmacology, version 2020.2) [27] by using the respective web services via the ‘Get Request’ and ‘XPath’ nodes in KNIME. Automated data access can be achieved by using predefined identifiers for targets, ligands (such as ligand structure, available bioactivities, or molecule names), biochemical assays, and so on.

The KNIME workflow for fetching ChEMBL data allows to map UniProt IDs of protein targets to target ChEMBL IDs and subsequent retrieval of ligand bioactivities and their respective structural information (here: canonical smiles), document ChEMBL IDs, and Pubmed IDs for the primary publication. A major challenge is the limited number of bioactivities (up to 1000 bioactivities) that are being fetched per single call. The KNIME workflow therefore has to be adopted to fetch all available data without manual intervention. The metanode that does the trick (termed ‘Get bioactivities per target’) works as follows:

- 1. A single XML file per target is downloaded and the number of bioactivities integrated within the < total_count > XML element is extracted.
- 2. The number of iterations needed to fetch all available bioactivities per target is calculated by dividing the number of bioactivities by 1000 and then rounding the result up (ceil() function in the ‘Math Formula’ node).
- 3. A recursive loop is used in order to process protein targets one-by-one.
- 4. A nested loop is used within a recursive loop where the API call is modified in a way that it dynamically changes the ‘offset’ parameter per each iteration. The ‘offset’ parameter determines the number of bioactivities that should be skipped before downloading the next portion of bioactivities for a given target. After the loop ends, all information needed is extracted from the collected XML files by the ‘Xpath’ node.

This procedure shall be illustrated on basis of an example: There are 2410 bioactivities for protein X available. Thus, three iterations are needed to fetch all data available for protein X if offset is set to 1000. Within each iteration, a column is appended to the table containing the API call with the corresponding offset parameter, i.e.

https://www.ebi.ac.uk/chembl/api/data/activity?target_chembl_id=CHEMBL5118&limit=1000&offset=0 (iteration#1).

https://www.ebi.ac.uk/chembl/api/data/activity?target_chembl_id=CHEMBL5118&limit=1000&offset=1000 (iteration#2).

https://www.ebi.ac.uk/chembl/api/data/activity?target_chembl_id=CHEMBL5118&limit=1000&offset=2000 (iteration#3).

At the end of the loop, 2410 bioactivities have been collected for protein X and these are processed as indicated in the description above.

**Fig. 5:**
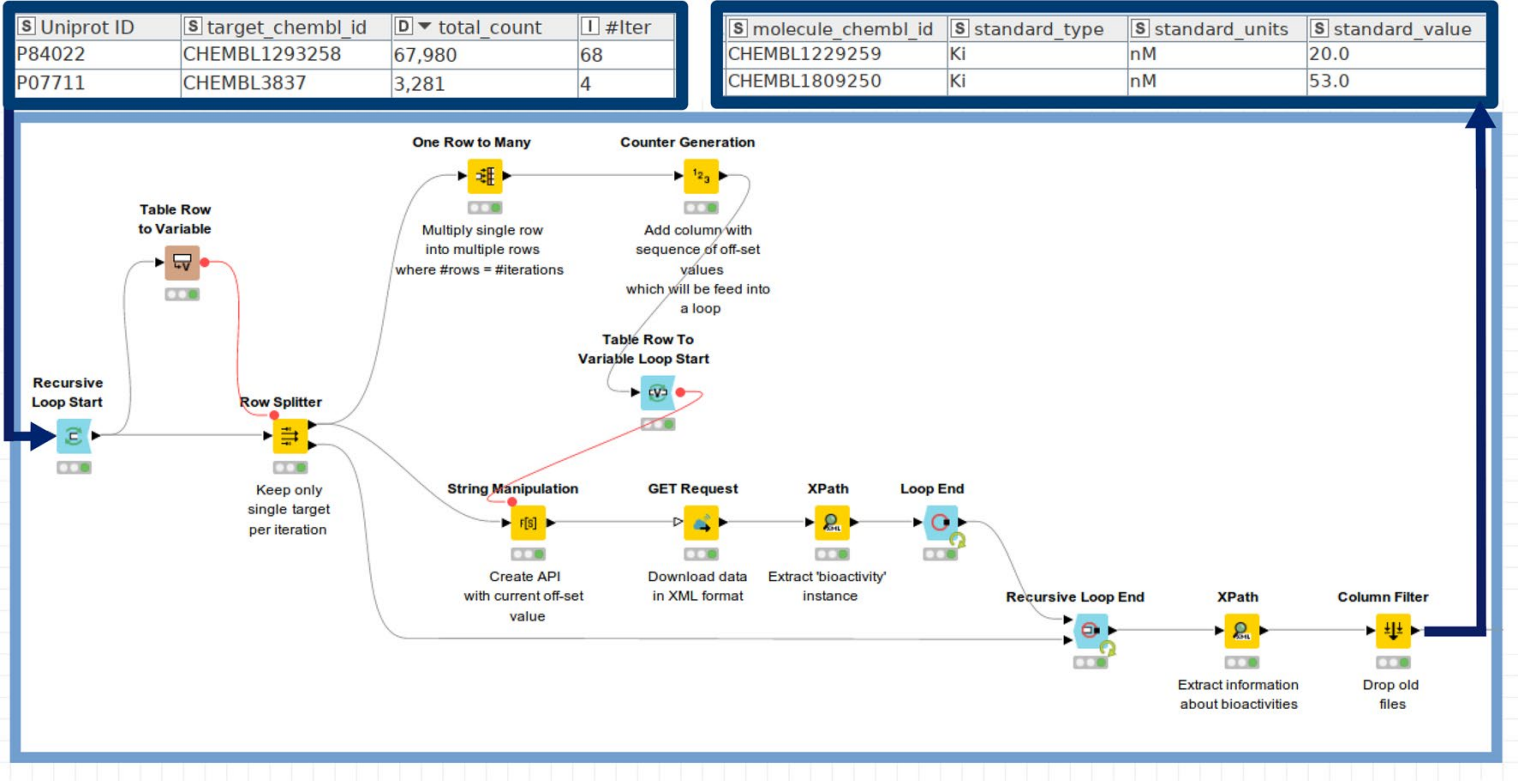
Nested recursive loop in order to fetch bioactivity data from ChEMBL (steps 3 and 4 of the metanode ‘Get bioactivities per target’): UniProt ID, Target ChEMBL ID, associated total count of available bioactivities, as well as the number of iterations needed for data download, are provided as input. The ‘Table Row to Variable’ node converts the respective UniProt ID into a variable and forwards it to the ‘Row Splitter’ Node. The table is thereafter split according to the UniProt ID, while the rest of the table is temporarily forwarded to the ‘Recursive Loop End’. The ‘One Row to Many’ node is used to multiply table rows on the basis of the number of iterations, as indicated in the ‘#Iter’ column. The ‘Counter Generation’ node is used to generate a column with the sequence of the offset values having an interval of 1000. The nested loop (initiated by the ‘Table Row To Variable Loop Start’ node) is used to create an API call (‘String Manipulation’ node) with the current value of the offset parameter. An API call is executed (‘GET Request’ node) and the bioactivity values are extracted accordingly (‘XPath’ node). The nested loop is running until all the offset values generated by the ‘Counter Generation’ node are applied. After the ‘Recursive Loop End’, the rest of the table from the second input port is passed back to the loop start and the workflow is repeated for the next protein target. As an output, each bioactivity data point (including activity comment, assay description, molecule ChEMBL ID, molecule general name, standard bioactivity type, standard unit, standard bioactivity value, standard relation, parent molecule ChEMBL ID, and document ChEMBL ID) is listed in the output table

Step 3 and 4 from the metanode ‘Get bioactivities per target’, as described above, are visually depicted in Fig. 5.

In case of PubChem, UniProt IDs are mapped to ‘PubChem Assay IDs’ (AID) in the first step. Further, AIDs are mapped to available compounds by ‘PubChem Compound ID’ (CID), including bioactivity measurements and associated PubMed IDs. Compound structures and names are retrieved in the next step. In some cases, compound names in PubChem are included in the form of molecule ChEMBL IDs. If this condition is true, the ChEMBL is additionally queried to download a compound name, if available.

In order to query IUPHAR data, the UniProt ID is mapped to the IUPHAR target ID. API calls have a specific syntax for accessing substrates, e.g.: http://www.guidetopharmacology.org/services/targets/2421/substrates and for accessing inhibitors, e.g.: http://www.guidetopharmacology.org/services/targets/2421/interactions,

where “2421” is an identifier for a specific target ID. Compound ID, PubMed ID, affinity, affinity type (corresponding to a certain end-point), and action (corresponding to a certain activity annotation) were retrieved by using the ‘JSON Path’ node. Retrieval of the ligand structural format is done by an additional API call on basis of the respective ligand ID.

Bioactivity values are converted to their negative logarithmic representation and binary labels (‘1’ for active and ‘0’ for inactive) are assigned on the basis of an activity cut-off. In this example, all compounds possessing a negative logarithmic value greater than 9 (i.e., < 1 nM) were labeled as ‘1’, while the rest was labeled as ‘0’.

After merging the output tables from ChEMBL, PubChem, and IUPHAR, the data is grouped to keep unique ligands per target and median values for binary activity labels (by using the ‘GroupBy’ node). In addition, only active ligands per target (label ‘1’) are kept and the final table is concatenated with ligand structures from PDB entries.

A prerequisite for merging ligand data from diverse sources is standardization of the molecular structures. A similar curation strategy like the one published by Gadaleta et al. [31] was applied:

- 1. Characters encoding stereoisomerism in SMILES format (@; \; /) are removed by using the ‘String Replacer’ node since for subsequent operations this information is not needed.
- 2. Salts are stripped by using the ‘RDkit Salt Stripper’ node. (This node works with pre-defined sets of different salts/salt mixtures by default. If requested, additional salt definitions can be forwarded to the node.)
- 3. Salt components are listed in the output table using the ‘Connectivity’ node (CDK plugin) followed by the ‘Split Collection Column’ node
- 4. The ‘RDKit Structure Normalizer’ node neutralizes charges and checks for atomic clashes, etc. Additional criteria for compound quality check can be adjusted in the ‘Advanced’ section of the node configuration.
- 5. The ‘Element Filter’ node keeps compounds containing the following elements only: H,C,N,O,F,Br,I,Cl,P,S).
- 6. InChI, InChiKey, and Canonical smiles formats are finally created from the standardized compounds.

**Fig. 6:**
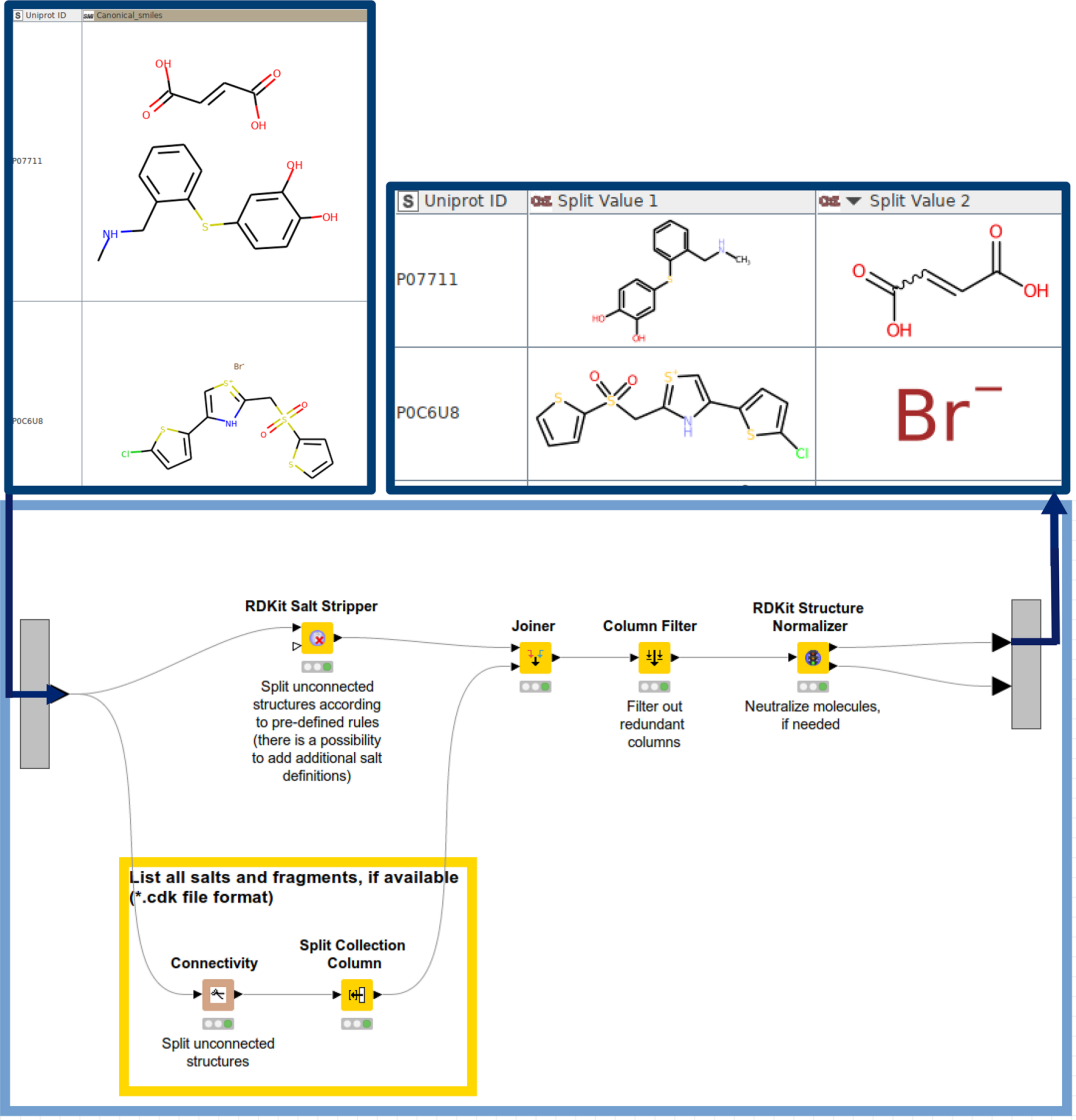
Standardization workflow used to strip salts and neutralize charges: The ‘RDKit’ Salt Stripper is used to split unconnected fragments according to predefined rules. The ‘Connectivity’ node (CDK) is used to list all the unconnected structures and list them in separate columns (‘Split Collection Column’ node). The output is joined together (‘Joiner’ node followed by the ‘Column Filter’ node to drop redundant columns). The ‘RDKit Structure Normalizer’ node is used to neutralize charges (if applicable). As an output of this part of the workflow, the neutralized molecule and the unconnected fragment(s) (if applicable) are listed in separate columns

Steps 2–4 are visually depicted in Fig. 6.

### 4. Step: Substructure searches to identify potentially interesting compounds for drug repurposing

Finally, the merged data sets are used to generate structural queries in SMARTS format in order to perform substructure searches in DrugBank (version 5.1.6, approx. 10,000 compounds, structures in SDF format are publicly available at https://www.drugbank.ca/releases/latest#structures) and in the COVID-19 antiviral candidate compound data set provided by the Chemical Abstracts Service (approx. 50,000 compounds, available upon request at https://www.cas.org/covid-19-antiviral-compounds-dataset).

**Fig. 7:**
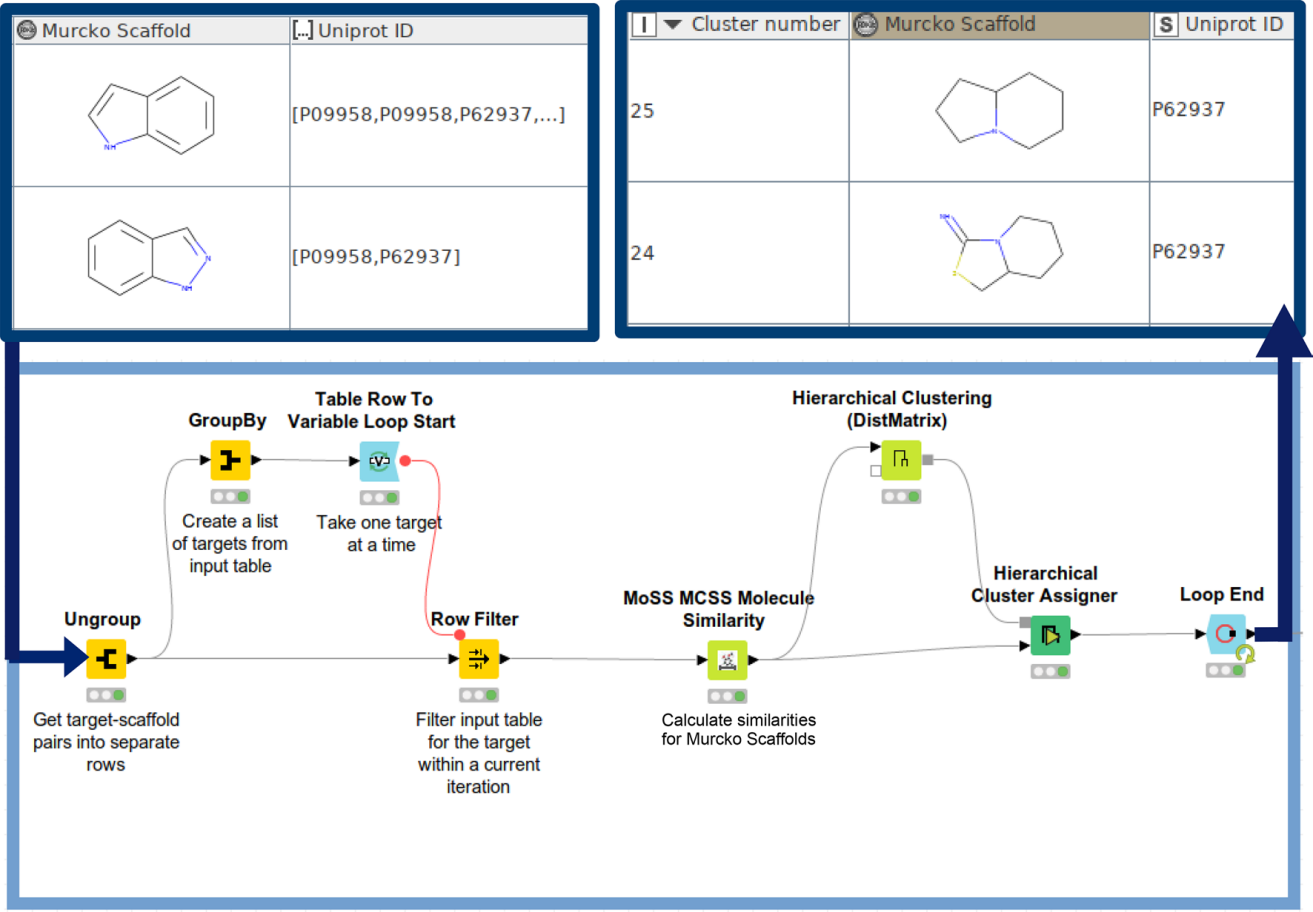
Hierarchical scaffold clustering in KNIME: Bemis-Murcko scaffolds and associated UniProt IDs (as a list) are forwarded as an input. The ‘Table Row To Variable Loop Start’ node forwards Bemis-Murcko scaffolds per respective target into the loop. Molecular distances are computed for the retained scaffolds (‘MoSS MCSS Molecule Similarity’ node) and hierarchical clusters are generated accordingly (the ‘Hierarchical Clustering [DistMatrix]’ node). Scaffolds are grouped into a specific group by applying the ‘Hierarchical Cluster Assigner’ node. The loop is then repeated for the remaining targets from the input table. The output table contains UniProt IDs, associated scaffolds, and cluster IDs

Bemis-Murcko scaffolds are extracted (‘RDKit Find Murcko Scaffolds’ node) in order to get a quick overview of the structural diversity of the curated data set. Scaffolds possessing too generic structures (i.e., a single aromatic ring) can be filtered out (by using the ‘RDKit Descriptors Calculator’ node in conjunction with the ‘Row Filter’ node) and remaining ones can be explored with respect to their structural similarity in the context of a certain target. This step is done by (1) calculating molecular distances (the ‘MoSS MCSS Molecule Similarity’ node), (2) hierarchical clustering (the ‘Hierarchical Clustering [DistMatrix]’ node), and (3) assigning a threshold (here: distance threshold = 0.5) for cluster assignment (the ‘Hierarchical Cluster Assigner’ node). The ‘MoSS MCSS Molecule Similarity’ node is used to calculate similarities between Murcko scaffolds by taking the size of their Maximum Common Substructure (MCS) as a similarity metric. Molecular similarities are then evaluated on the basis of a distance matrix. The respective part of the workflow is depicted in Fig. 7.

**Fig. 8:**
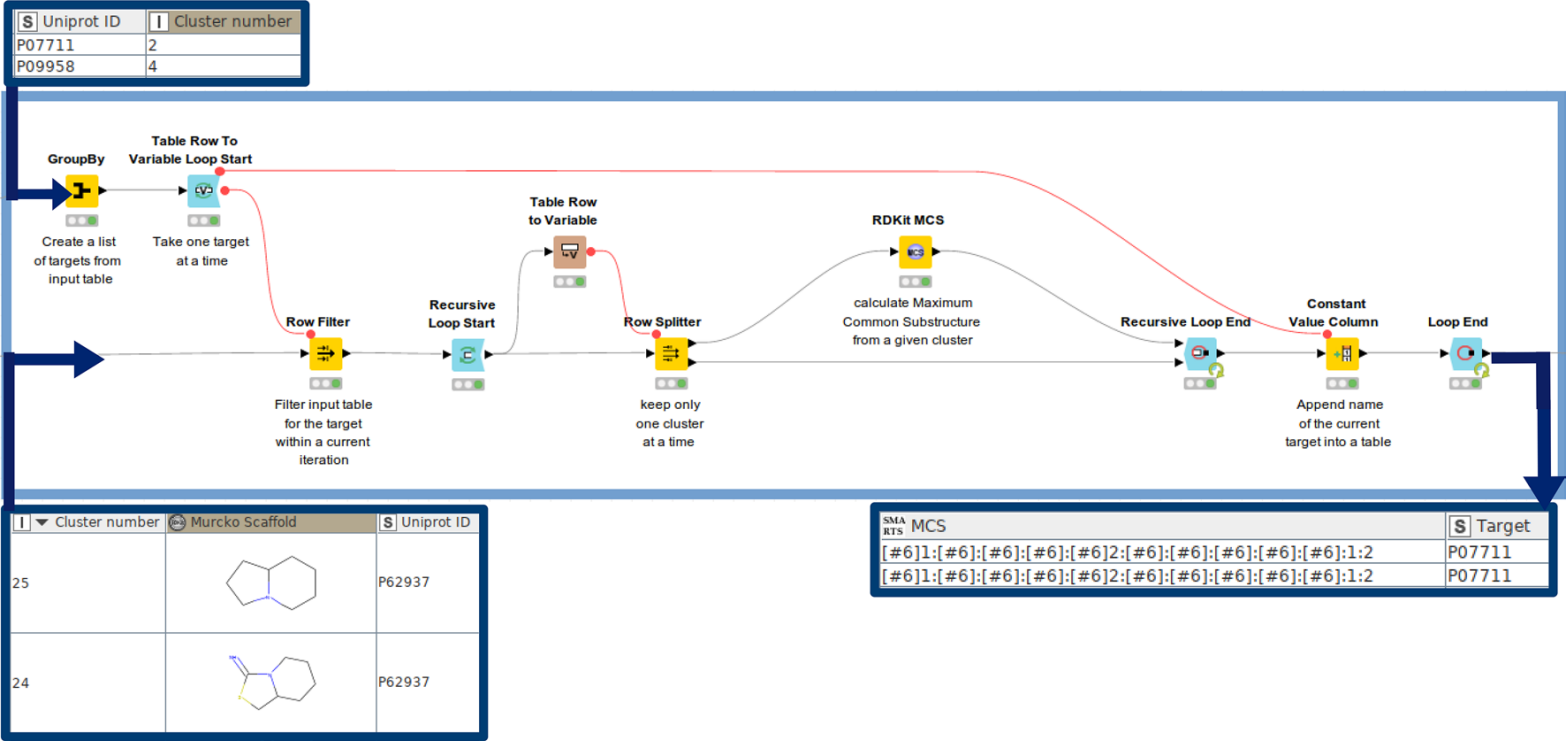
Looping through scaffold clusters and generating a maximum common substructure for a given cluster: The ‘Table Row To Variable Loop Start’ accepts a single target per iteration and the ‘Recursive loop start’ takes a single cluster (indicated by the ‘Cluster number’ column) per iteration. From the scaffolds grouped into a single cluster their maximum common substructure (the ‘RDKit MCS’ node) is calculated. The output table contains the target as a UniProt ID and associated substructures in SMARTS format

Next, looping over distinct clusters of associated Bemis-Murcko scaffolds for a respective target is done in order to create a maximum common substructure (the ‘RDKit MCS’ node) from all associated scaffolds belonging to a respective cluster. Recursive loops are extensions to regular loops which can be used in conjunction with a ‘Row Splitter’ node to separate the current row from the rest of the table. After termination of the current iteration, the rest of the table is forwarded to the loop start and the next row is used for the subsequent iteration (see Fig. 8). Generated substructures for a certain target are appended to the output table in SMARTS format.

**Fig. 9:**
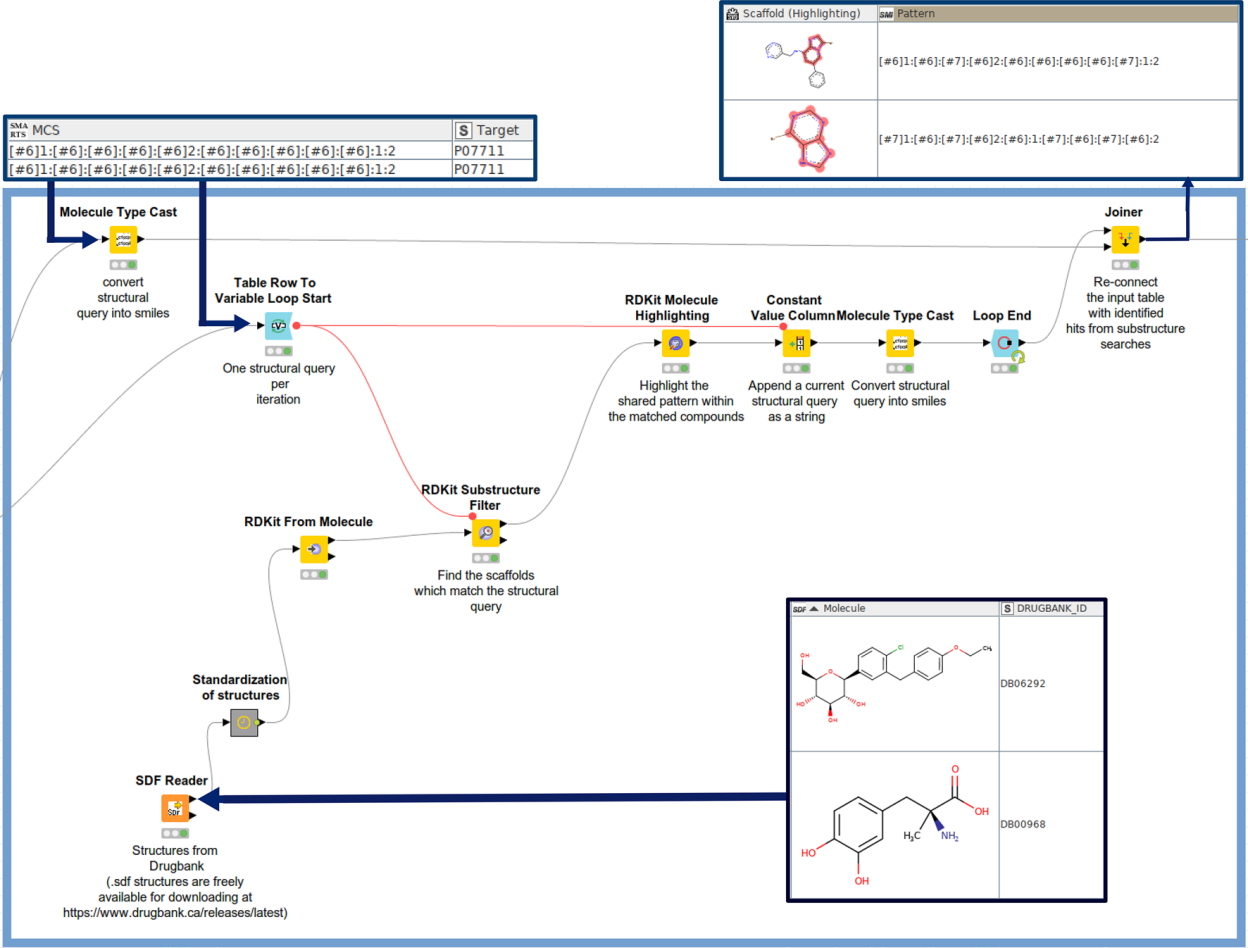
Automated substructure searches in KNIME: The loop iterates through the input substructure queries (in SMARTS) to find hits in DrugBank (input data set includes molecules in SDF format, DrugBank IDs, and associated content). The structures from DrugBank are standardized (‘Standardization of structures’ metanode), and then subjected to the ‘RDKit Substructure Filter’ node to perform substructure searches. The SMARTS query is forwarded to the input as a variable. Detected substructures are highlighted by the ‘RDKit Molecule Highlighting’ node. The output table contains identified hits (molecule names, associated targets, SMARTS keys, chemical structures), and highlighted substructures in SVG format

For the substructure searches in DrugBank and the CAS data set loops are being used as well (Fig. 9). The ‘Table Row To Variable Loop Start’ forwards each substructure as a query to the ‘RDKit Substructure Filter’ node as a flow variable which then examines whether a particular substructure is contained in the data sets from DrugBank or CAS. Extracted compounds are being forwarded to the ‘RDKit molecule highlighting’ node which visualizes the highlighted substructure within the respective compounds.

### Software

KNIME workflows were built in KNIME version 4.1.2. The KNIME workflows are freely available from GitHub (https://github.com/AlzbetaTuerkova/Drug-Repurposing-in-KNIME). The published workflow can be either used as a single pipeline, or as multiple stand-alone workflows (1) to gather data from PDB, (2) to retrieve ligand bioactivities from ChEMBL, PubChem, and IUPHAR, and (3) to perform substructure searches, by providing the needed data input, respectively.

## Results and discussion

In this contribution, a semiautomatic KNIME workflow for drug repurposing based on publicly available structural- and bioactivity-ligand data is presented. The pipeline includes automatic mapping of UniProtKB entries and PDB via cross-referencing, programmatic data access via the data sources’ web services (exemplified for ChEMBL, PubChem, and IUPHAR), fully automatic data curation (including data integration, chemical data standardization, removal of duplicates, and cut off setting for assigning activity labels), the identification of common structural patterns in SMARTS format, and substructure searches (here in DrugBank and the CAS data set of antiviral drugs) in order to identify interesting compounds for further investigations. The drug repurposing pipeline developed here is showcased by applying it on one rare disease as well as one new disease: Glucose Transporter Type 1 (GLUT-1) deficiency syndrome and COVID-19.

### Retrieval of COVID-19 data

The Universal Protein Resource KnowledgeBase (UniProtKB) is a freely accessible database for protein sequence and annotation data. The UniProt ID (e.g., P59596, P59637, P0C6X7) is a protein identifier which can be used to retrieve comprehensive information about a given protein, including protein names and synonyms, taxonomy, function, cellular localization, available three-dimensional structures, as well as cross-references to other databases. Cross-referenced databases include (but are not limited to) sequence databases (e.g. GenBank [32], CCDS [33]), 3D structure databases (e.g., Protein Data Bank [29], ModBase [34], SWISS-MODEL-Workspace [35]), protein–protein interaction databases (e.g., Biogrid [36], IntAct [37], STRING [38]), and chemistry databases (e.g., BindingDB, [39] ChEMBL, [4] DrugBank [7]). In a first instance, content from a pre-release UniProt web page (available at https://covid-19.uniprot.org/uniprotkb?query=*) was used as an input for the data mining pipeline to gather and analyze data for proteins potentially interesting for the treatment of infections with human SARS-CoV-2 (53 proteins, Additional file 1: Table S1). As seen from Additional file 1: Table S1, available protein templates include 14 SARS-CoV-2, 15 SARS-CoV, and 24 structures with origin Homo Sapiens.

Listed UniProt IDs were used to retrieve protein structures stored in PDB (1084 structures, 953 unique structures). From these sources, 151 unique ligands could be extracted, yielding 87 unique Murcko scaffolds. From the orthogonal approach—the automatic gathering of ligand bioactivity data from ChEMBL, PubChem, and IUPHAR via its webservices—3951 unique ligands with (median) activity value < 1 nM were identified (2555 unique Murcko scaffolds).

As an alternative solution for generating a list of targets associated to COVID-19, 55 human protein targets with the association score of at least 0.99 were retrieved from the Open Targets Platform (see Additional file 1: Table S2). Interestingly, the interleukin-6 receptor subunit alpha (UniProt ID P08887) was identified as a sole target which was also listed at the UniProt pre-release web page. Such a different constitution of the input data between the UniProt pre-release webpage and the Open Targets Platform could be explained by the fact that target-disease association scores in Open Targets are based on a cumulative score collecting different sources of evidence (such as genetic associations, somatic mutations, drugs available in ChEMBL, pathways & system biology, RNA expression data, text mining, animal models). However, in the case of COVID-19 to date only association scores for evidence from drugs in ChEMBL and text mining are available, which restricts the highly scored targets to the ones already described in literature. The approach did not allow for prioritization of, e.g., ACE2 receptor, as its association score possesses a value of only 0.11 in the Open Targets Platform (accessed Sept. 2020). This use case might illustrate the ultimate benefit when combining protein-disease association data from various independent sources.

Listed targets originating from the Open Target platform have become a source of multiple PDB structures (571 structures, 502 unique structures). In total, 85 unique ligands could be extracted, (45 unique Bemis-Murcko scaffolds). By applying integrative mining of bioactivity data from public databases, 3207 unique ligands with (median) activity value < 1 nM were fetched (1710 unique Bemis-Murcko scaffolds).

The highly ranked targets (based on the number of retrieved compounds) from either resource are listed in Table 1.

**Table 1:** Number of compounds available from different data sources (PDB, ChEMBL, IUPHAR, PubChem) for the five top-ranked protein targets retrieved from both the UniProt pre-release web page and the Open Targets Platform

Target shortcut	Target source	PDB	ChEMBL	IUPHAR	PubChem	# Unique active compounds
PPIA_HUMAN	UniProt pre-release	57	2	1	3123	3183
CATL1_HUMAN	UniProt pre-release	25	38	4	946	1003
ITAL_HUMAN	UniProt pre-release	13	94	2	550	564
FURIN_HUMAN	UniProt pre-release	4	10	1	448	463
R1AB_CVHSA	UniProt pre-release	37	187	0	47	227
GABRG2_HUMAN	Open Targets Platform	152	0	677	2	831
MMP13_HUMAN	Open Targets Platform	319	3	80	28	430
GABRB1_HUMAN	Open Targets Platform	121	0	166	0	287
DPP4_HUMAN	Open Targets Platform	140	2	29	14	185
GABRA1_HUMAN	Open Targets Platform	3	4	172	2	181

### Analysis of COVID-19 data sets

Numbers of unique compounds per individual COVID-19 drug target that could be fetched from the different data sources are listed in Additional file 1: Tables S3 and S4. In case of targets retrieved from the UniProt pre-release web page (Additional file 1: Table S3), PubChem is the predominant source of ligands (9751 unique compounds). At the other end of the scale, IUPHAR provides 19 unique compounds only. Inspecting the origin of data for the respective protein targets, it becomes apparent that the ligand information for human SARS-CoV-2 solely originates from PDB structures (see Additional file 1: Table S3, entries ending with “_SARS2”). Notably, the majority of structures for SARS-CoV-2—such as PDB IDs 6W4B [40], 6Y2E, or 6Y2G for replicase polyprotein 1a [41] were refined via molecular replacement based on the homology to SARS-COV. It therefore seems to be beneficial to integrate data from diverse sources, especially including PDB as a source for most up-to-date compound information.

Across all data sources, the largest number of ligand bioactivity measurements (in case of targets from UniProt pre-release web page) was gathered for human peptidyl-prolyl cis–trans isomerase A (UniProt ID P62937; 3183 unique compounds), followed by human procathepsin L (UniProt ID P07711; 1003 unique compounds), human integrin alpha-L (UniProt ID P20701; 564 unique compounds), human furin (UniProt ID P09958; 463 unique compounds), SARS replicase polyprotein 1ab (UniProt ID P0C6X7; 227 unique compounds), human angiotensin-converting enzyme 2 (ACE2; UniProt ID Q9BYF1; 172 unique compounds), SARS replicase polyprotein 1a (UniProt ID P0C6U8; 141 unique compounds), and human mothers against decapentaplegic homolog 3 (UniProt ID P84022; 71 unique compounds). For other potential COVID-19 targets, only a neglectable number of compounds was retrieved. The ACE2 receptor is considered a relevant therapeutic target due to its interaction with spike glycoprotein of coronaviruses when entering host cells [42]. Replicase polyproteins 1a and 1ab are attractive targets to treat COVID-19 given their crucial role in replication and transcription of viral RNAs [43]. A current study has suggested a potential role of integrins as alternative receptors for SARS-CoV-2, as the spike glycoprotein contains an integrin-binding motif [44].

From the data retrieved from the Open Targets Platform, a complete list of unique compounds per individual target is included in Additional file 1: Table S4. The highest number of unique compounds was retrieved for gamma-aminobutyric acid type A receptor subunit gamma 2 (UniProt ID P18507; 831 unique compounds). In general, different gamma-aminobutyric receptor subunits have been ranked high in terms of the number of gathered compounds.

### COVID-19 case: substructure searches in external data sets

Chemical (molecular) similarity is a traditional concept in the field of cheminformatics [45]. It is used to identify structural analogs which might exert similar biological action on similar biological targets [46]. Common cheminformatics similarity approaches are based on the global similarity of a molecule. For example, fingerprint-based descriptors are used to evaluate compound similarity by quantifying the presence/absence of the specific structural features (e.g., distinct functional groups in a molecule). On the contrary, molecular graph-based methods do capture a specific molecular topology and hence account for the local similarity of molecules [47]. Graph-based methods are therefore a robust tool to, e.g., distinguish between different structural isomers (such as n-pentane and dimethylpropane). Here, Maximum Common Substructures (MCS) of a compound collection were used as structural keys for detecting new potential drug candidates. Such substructure searches are especially useful for drug repositioning strategies, since they more likely capture the local similarity of chemical compounds and therefore allow for more flexibility than global similarity measures (especially if there are large differences of the size of compounds that are being compared).

In a first instance, the Bemis-Murcko scaffold for identified ligands was extracted. For each target, scaffolds were grouped into hierarchical clusters by considering their Maximum Common Substructure (MCS) as a measure of similarity. Afterwards, looping in KNIME was applied to generate one MCS (in SMARTS) per cluster (and target). For details see the Methods Section. In total, 257 distinct MCSs were calculated. A complete list of MCSs can be found in Additional file 1: File S1.

Structural queries generated in the previous step helped identify 7836 compounds from DrugBank and 36,521 compounds from the CAS data set. A complete list of hits found by the substructure searches is provided in Additional file 1: File S2 (DrugBank) and Additional file 1: File S3 (CAS data set). Out of those hits, 135 compounds were retrieved from both DrugBank and the CAS data set (Additional file 1: File S4) and were identified on basis of 18 distinct MCSs (Table 2). Identified MCSs can be combined into five separate clusters (Table 2): (1) Hits identified on basis of the open-chain structural keys (59 hits), (2) Nucleoside/nucleotide analogs (53 hits), (3) miscellaneous, which contain ubiquitous substructures (22 hits), (4) cyclopropane-containing hits (3 hits), and (5) adamantane derivatives (3 hits). Supplementary Figure S1 shows examples of identified hits for the most pronounced clusters. It has to be noted, that the searches do also retrieve compounds that were part of the list of structural queries that were used as an input. For example, remdesivir was rediscovered as part of the substructure searches but it was also included in the original input file. However, for the COVID-19 use case, only less than 2% of hits (820 out of 43,259 compounds) were already part of the input query file.

**Table 2:** COVID-19 case: Five clusters of enriched Maximum Common Substructures which were retrieved from DrugBank and the CAS data set

Cluster number	Maximum Common Substructure	SMARTS String	# Hits	Targets
1		[#6](:[#7]:[#6]:[#7]-,:[#6]-,:[#6]-[#6]-[#6]):[#6]	53	PPIA_HUMAN
		[#6](-,:[#6]-,:[#6]-[#6]-[#6] = [#6]-[#6])-,:[#6]-,:[#6]	5	PPIA_HUMAN
		[#6]:,-[#6]-,:[#7]-,:[#6]:,-[#6]:,-[#6]:,-[#6]:[#6]:[#6]	1	PPIA_HUMAN
2		[#6]1:[#7]:[#6]:[#7]:[#6]2:[#6]:1:[#7]:[#6]:[#7]:2-[#6]1-[#8]-[#6]-[#6]-[#6]-1	36	R1AB_SARS2
		[#7]1:[#6]:[#7]:[#6]2:[#6]:1:[#7]:[#6]:[#7]:[#6]:2	11	PPIA_HUMAN
		[#6]1:[#7]:[#6]:[#7](-[#6]2-[#8]-[#6]-[#6]-[#6]-2):[#7]:1	3	PPIA_HUMAN
		[#6]1:[#7]:[#6]:[#7]:[#6](:[#6]:1):[#7](:[#6])-[#6]1-[#8]-[#6]-[#6]-[#6]-1	2	PPIA_HUMAN
		[#6]1(-[#6]2:[#6]:[#6]:[#6]3:[#6]:[#7]:[#6]:[#7]:[#7]:2:3)-[#8]-[#6]-[#6]-[#6]-1	1	R1AB_SARS2
3		[#7]1-[#6]-[#6]-[#7]-[#6]-[#6]-1	8	R1AB_SARS2
		[#6]-:[#6]-:[#6](-[#6](= [#8])-[#7]-[#6](:-[#6]:-[#6]):-[#6]:-[#6])-:[#6]-:[#6]	4	R1AB_SARS2
		[#6]1:[#6]:[#6]:[#6]2:[#6](:[#6]:1)-[#8]-[#6]-[#8]-2	3	R1AB_SARS2
		[#6]1:[#7]:[#6]:[#6]:[#6]:[#6]:1	3	PPIA_HUMAN
		[#6](-[#6]-[#6]1-[#6]-[#6]-[#7]-[#6]-1 = [#8])-[#7]-[#6](= [#8])-[#6]	2	R1A_SARS, R1AB_SARS
		[#6](-[#6]-[#6]1-[#6]-[#6]-[#7]-[#6]-1 = [#8])-[#7]-[#6](= [#8])-[#6]-[#6]-[#6]1:[#6]:[#6]:[#6]:[#6]:[#6]:1	2	R1A_SARS, R1AB_SARS
4		[#6](-[#7]-[#6](= [#8])-[#6]1-[#6](-[#6]-[#6]-[#7]-1-[#6](= [#8])-[#6]-[#7]-[#6] = [#8])-[#6])-[#6]-[#6](= [#8])-[#7]-[#6]1-[#6]-[#6]-1	1	R1AB_SARS2
		[#6](-[#7]-[#6](= [#8])-[#6]1-[#6]2-[#6]-[#6]-[#6]-[#6]-2-[#6]-[#7]-1)-[#6]-[#6](= [#8])-[#7]-[#6]1-[#6]-[#6]-1	1	R1AB_SARS2
		[#7]1-[#6]-[#6]2-[#6](-[#6]-1-[#6](= [#8])-[#7]-[#6]-[#6]-[#6]1-[#6]-[#6]-[#6]-1)-[#6]-2	1	R1AB_SARS2
5		[#6]12-[#6]-[#6]3-[#6]-[#6](-[#6]-1)-[#6]-[#6](-[#6]-2)-[#6]-3	3	PPIA_HUMAN

The structural fragment, SMARTS string, the number of identified hits, and the protein target(s) for which these hits have been found, are given

### GLUT-1 deficiency syndrome

Glucose transporter type 1 (GLUT1) deficiency syndrome is characterized by the impairment of glucose transport that might be attributed to mutations in the SLC2A1 gene. Indeed, glucose transporter 1 (GLUT1, encoded by SLC2A1 gene) has been identified as a sole target associated with this disease (with an association score of 1.00 in the Open Targets Platform). Glucose transporter 1 (GLUT-1) is a member of the SLC2A transporter subfamily, being ubiquitously expressed in different tissues, including fetal tissues, mammary glands, placenta, brain, or epithelial cells [48]. GLUT-1 is an essential transmembrane protein for basal glucose uptake.

Symptoms of GLUT1 deficiency syndrome are predominantly seizures, epilepsy and cognitive deficit. GLUT-1 deficiency syndrome is treatable via ketogenic diet [49]. Furthermore, several drugs (e.g., Triheptanoin, DrugBank ID DB11677) have been tested in clinical trials for their efficacy. Up to now, no drug candidate has been found to become an effective treatment for GLUT-1 deficiency syndrome. This neurologic disorder belongs to the group of rare diseases and therefore represents an interesting case study for our drug repurposing pipeline.

### Retrieval of GLUT-1 data

By mapping GLUT-1 retrieved from the Open Target Platform to UniProt IDs, protein structures stored in PDB (4 unique structures) have been fetched. From these sources, 4 unique ligands could be extracted, yielding 3 unique Bemis-Murcko scaffolds. Integrative mining of bioactivity data delivered 653 unique compounds from ChEMBL (394 unique Murcko scaffolds), 243 unique compounds from PubChem (115 unique Murcko scaffolds), and 2 unique compounds from IUPHAR (2 unique Murcko scaffolds) with activity < 1 μM. The threshold for activity label assignment was adopted due to the specific activity range characteristic for membrane transporters [50, 51].

### GLUT-1 case: substructure searches in DrugBank

**Fig. 10:**
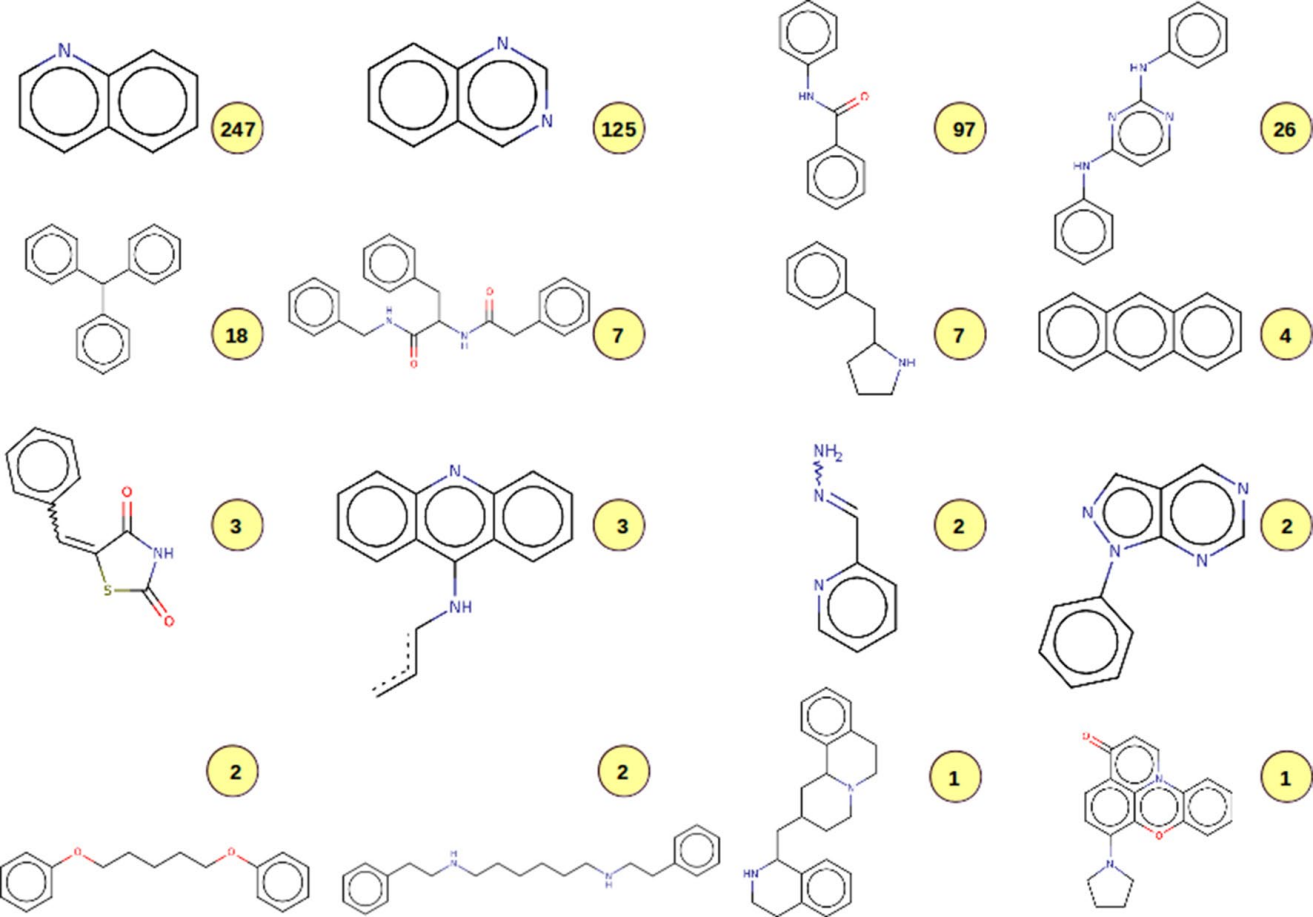
Enriched structural queries retrieved for the target GLUT-1. The numbers in yellow circles indicate the number of identified hits per given structural query

Hierarchical clustering of available Murcko scaffolds delivered 94 fragments used for substructure searches in DrugBank. 18 different fragments (depicted in Fig. 10) have been enriched in 539 unique compounds retrieved from DrugBank (Additional file 1: File S5). 14% of the retrieved hits (28 out of 200 compounds in total) were already part of the input query file and were therefore rediscovered as part of the substructure searches.

It appears interesting that most of the identified hits do contain a quinoline (n = 247 hits) or quinazoline (n = 125) scaffold. These heterocyclic compounds are broadly pharmacologically active [51, 52]. Interestingly, quinoline/quinazoline analogs have been inspected to become promising anticonvulsant agents, as indicated in different studies [53–55]. Since 90% of all patients with this syndrome also develop frequent seizures (https://medlineplus.gov/genetics/condition/glut1-deficiency-syndrome/) these classes of compounds could be interesting for future investigations on GLUT-1 deficiency syndrome.

### Experiences when using the workflow in the classroom

The workflow described herein has been used in the summer semester 2020 (April 20–24) in the framework of the course “Experimental Methods in Drug Discovery and Preclinical Drug Development'' which is part of the English-language Master’s Degree Program *Drug Discovery and Development* at the University of Vienna (https://drug-dd.univie.ac.at/). Due to the requirements of social distancing caused by the COVID-19 pandemic, this course was conceptualized as a virtual classroom. The students have attended online sessions, in which the authors of this manuscript have explained the various steps of the workflow. Tutorials and the different parts of the workflow (available at https://github.com/AlzbetaTuerkova/Drug-Repurposing-in-KNIME) have been handed out daily in order to not overwhelm the students. On the last day of this 5-days course, each student had to select one of the hits retrieved by the substructure search and dedicate some time to literature searches. Finally, every student submitted a report summarizing what is known about the selected compound and its potential usefulness for COVID-19 treatment (according to what was known in April 2020). Based on the feedback that was provided by students after the course was finalized, the pace of teaching was evenly distributed over the course schedule. The only exception was the step when bioactivity data was retrieved from ChEMBL and PubChem on day 3 of the course. Specifically, some students found it difficult to grasp the essence of the application and execution of the recursive and/or nested loops. In conclusion, the course did provide insights into a variety of KNIME nodes, which can be exploited further for future drug discovery applications.

## Summary and conclusions

In this educational paper, we are describing a semi-automatic KNIME workflow for ligand-based in silico drug repurposing. The consecutive data mining steps include integration, curation, and analysis of bioassay data from the open domain for specific targets of interest, as well as the generation of structural queries for automated substructure searches in collections of approved, withdrawn, and/or experimental drugs. Targeted access of data through APIs has been implemented at several stages of the KNME workflow. Incorporation of API calls into KNIME allows repeating the whole procedure in an automated fashion, e.g., when new data is becoming available. As a consequence of the current COVID-19 pandemic, the cheminformatics analyses performed as a use case herein was tailored to ligand and protein data currently available for drug repurposing strategies in the framework of this life-threatening disease. As a side effect of analyzing the data, we are providing insights into enriched chemical substructures for proposed drug targets of SARS-CoV-2. In addition, the workflow has been used to detect data coverage and enriched clusters for the treatment of a rare disease, GLUT-1 deficiency syndrome. The material has been used successfully for teaching undergraduate students the use of programmatic data access via KNIME workflows and subsequent data analysis steps. The workflows, tutorials, and the information gained on COVID-19 and GLUT-1 data are freely available to the scientific community for follow-up studies or may be tailored to specific needs of other use cases (available at https://github.com/AlzbetaTuerkova/Drug-Repurposing-in-KNIME).

## Supplementary information

**Additional file 1.** Supplementary Information—(1) a list of drug targets with potential interest for treatment of COVID-19, available from https://covid-19.uniprot.org/uniprotkb?query=* and (2) from the Open Targets Platform, (3) number of unique ligands gathered from PDB, ChEMBL, PubChem, and IUPHAR for COVID-19 targets from UniProt pre-release web page and (4) from the Open Targets Platform (5) examples of identified drugs with the highlighted structural query, and (6) description of the supplementary data files.

## Abbreviations

API: Application Programming Interface
KNIME: Konstanz Information Miner
CDK: Chemistry Development Kit
UniProtKB: The Universal Protein Resource KnowledgeBase
COVID-19: Coronavirus Disease 2019
SARS-CoV-2: Severe Acute Respiratory Syndrome Coronavirus 2
PDB: Protein Data Bank
NMR: Nuclear Magnetic Resonance
Cryo-EM: Cryo-Electron Microscopy
RCSB: Research Collaboratory for Structural Bioinformatics
AID: Assay ID
CID: Compound ID
CAS: Chemical Abstract Service
MCS: Maximum Common Substructure
PCA: Principal Component Analysis
LabuteASA: Labute’s Accessible Surface Area
SMR: Molecular Refractivity
TPSA: Topological Polar Surface Area
GLUT-1: Glucose Transporter Type 1
URL: Uniform Resource Locator
