# Supplementary material for: A ligand-based computational drug repurposing pipeline using KNIME and Programmatic Data Access: case studies for rare diseases and COVID-19
Source: J Cheminform. 2020 Nov 25;12:71. doi: 10.1186/s13321-020-00474-z (PMC7686838; doi:10.1186/s13321-020-00474-z)
Supplement: Supplementary file 1 — Additional file 1. Supplementary Information—(1) a list of drug targets with potential interest for treatment of COVID-19, available from https://covid-19.uniprot.org/uniprotkb?query=* and (2) from the Open Targets Platform, (3) number of unique ligands gathered from PDB, ChEMBL, PubChem, and IUPHAR for COVID-19 targets from UniProt pre-release web page and (4) from the Open Targets Platform (5) examples of identified drugs with the highlighted structural query, and (6) description of the supplementary data files. [file 13321_2020_474_MOESM1_ESM.pdf]

A Ligand-based Computational Drug Repurposing Pipeline using KNIME and Programmatic Data Access: Cases Studies for Rare Diseases and COVID-19

Alzbeta Tuerkova and Barbara Zdrazil**

University of Vienna, Department of Pharmaceutical Chemistry, Division of Drug Design and Medicinal Chemistry, Althanstraße 14, A-1090 Vienna, Austria.

Table of Contents

Supplementary Table S1 .... S2

Supplementary Table S2 .... S4

Supplementary Table S3 .... S7

Supplementary Table S4 .... S9

Supplementary Figure S1 .... S12

Description of Supplementary Data Files S13

Supplementary Table S1: Protein targets with potential interest for treatment of COVID-19 (available from https://covid-19.uniprot.org/uniprotkb?query=*), retrieved August 2020.

UniProt ID	Target Name	Organism	Target Shortcut
O15393	Transmembrane protease serine 2	Homo sapiens	TMPS2_HUMAN
Q92499	ATP-dependent RNA helicase DDX1	Homo sapiens	DDX1_HUMAN
Q9BYF1	Angiotensin-converting enzyme 2	Homo sapiens	ACE2_HUMAN
O43765	Small glutamine-rich tetratricopeptide repeat	Homo sapiens	SGTA_HUMAN
P20701	containing protein alpha	Homo sapiens	ITAL_HUMAN
P35232	Integrin alpha-L	Homo sapiens	PHB_HUMAN
P84022	Prohibitin	Homo sapiens	SMAD3_HUMAN
Q8N3R9	Mothers against decapentaplegic homolog 3	Homo sapiens	MPP5_HUMAN
Q99623	MAGUK p55 subfamily member 5	Homo sapiens	PHB2_HUMAN
P05231	Interleukin-6	Homo sapiens	IL6_HUMAN
P07711	Procathepsin L	Homo sapiens	CATL1_HUMAN
P08887	Interleukin-6 receptor subunit alpha	Homo sapiens	IL6RA_HUMAN
P09958	Furin	Homo sapiens	FURIN_HUMAN
P35613	Basigin	Homo sapiens	BASI_HUMAN
P40189	Interleukin-6 receptor subunit delta	Homo sapiens	IL6RB_HUMAN
P52292	Importin subunit alpha-1	Homo sapiens	IMA1_HUMAN
P62937	Peptidyl-prolyl cis-trans isomerase A	Homo sapiens	PPIA_HUMAN
Q10589	Bone marrow stromal antigen 2	Homo sapiens	BST2_HUMAN
Q16552	Interleukin-17A	Homo sapiens	IL17_HUMAN
Q8NAC3	Interleukin-17 receptor C	Homo sapiens	I17RC_HUMAN
Q8NHX9	Two pore calcium channel protein 2	Homo sapiens	TPC2_HUMAN
Q96F46	Interleukin-17 receptor A	Homo sapiens	I17RA_HUMAN
Q96PD4	Interleukin-17F	Homo sapiens	IL17F_HUMAN
Q9Y2I7	1-phosphatidylinositol 3-phosphate 5-kinase	Homo sapiens	FYV1_HUMAN
Q99623	Prohibitin-2	SARS COV	R1A_CVHSA
P0C6U8	Replicase polyprotein 1a	SARS COV	R1AB_CVHSA

P0C6X7	Replicase polyprotein 1ab	SARS COV-2	R1A_SARS2
P0DTC1	Replicase polyprotein 1a	SARS COV-2	R1AB_SARS2
P0DTD1	Replicase polyprotein 1ab	SARS COV-2	SPIKE_SARS2
P0DTC2	Spike glycoprotein	SARS COV	SPIKE_CVHSA
P59594	Spike glycoprotein	SARS COV	NCAP_CVHSA
P59595	Nucleoprotein	SARS COV	AP3A_CVHSA
P59632	Protein 3a	SARS COV	NS7A_CVHSA
P59635	Protein 7a	SARS COV	VEMP_CVHSA
P59637	Envelope small membrane protein	SARS COV	VME1_CVHSA
P59596	Membrane protein	SARS COV	NS3B_CVHSA
P59633	Non-structural protein 3b	SARS COV-2	AP3A_SARS2
P0DTC3	Protein 3a	SARS COV-2	VME1_SARS2
P0DTC5	Membrane protein	SARS COV-2	NS7A_SARS2
P0DTC7	Protein 7a	SARS COV-2	NCAP_SARS2
P0DTC9	Nucleoprotein	SARS COV	NS6_CVHSA
P59634	Non-structural protein 6	SARS COV	ORF9B_CVHSA
P59636	Protein 9b	SARS COV-2	VEMP_SARS2
P0DTC4	Envelope small membrane protein	SARS COV-2	NS6_SARS2
P0DTC6	Non-structural protein 6	SARS COV-2	ORF9B_SARS2
P0DTD2	Protein 9b	SARS COV	NS7B_CVHSA
Q7TFA1	Protein non-structural 7b	SARS COV	NS8B_CVHSA
Q80H93	Non-structural protein 8b	SARS COV-2	NS8_SARS2
P0DTC8	Non-structural protein 8	SARS COV-2	Y14_SARS2
P0DTD3	Uncharacterized protein 14	SARS COV-2	NS7B_SARS2
P0DTD8	Protein non-structural 7b	SARS COV	NS8A_CVHSA
Q7TFA0	Protein non-structural 8a	SARS COV	Y14_CVHSA
Q7TLC7	Uncharacterized protein 14	SARS COV-2	A0A663DJA2_SARS2
A0A663DJA2	ORF10 protein		

Supplementary Table S2: Protein targets with potential interest for treatment of COVID-19 retrieved from the Open Targets Platform; retrieved in September 2020.

Uniprot ID	Target Name	Organism	Target Shortcut
P34903	gamma-aminobutyric acid type A receptor subunit alpha3	Homo Sapiens	GABRA3_HUMAN
P14867	gamma-aminobutyric acid type A receptor subunit alpha1	Homo Sapiens	GABRA1_HUMAN
P35354	prostaglandin-endoperoxide synthase 2	Homo Sapiens	PTGS2_HUMAN
O95069	potassium two pore domain channel subfamily K member 2	Homo Sapiens	KCNK2_HUMAN
O00591	gamma-aminobutyric acid type A receptor subunit pi	Homo Sapiens	GABRP_HUMAN
P23219	prostaglandin-endoperoxide synthase 1	Homo Sapiens	PTGS1_HUMAN
P62877	ring-box 1	Homo Sapiens	RBX1_HUMAN
P57789	potassium two pore domain channel subfamily K member 10	Homo Sapiens	KCNK10_HUMAN
Q9H4B7	tubulin beta 1 class VI	Homo Sapiens	TUBB1_HUMAN
P78334	gamma-aminobutyric acid type A receptor subunit epsilon	Homo Sapiens	GABRE_HUMAN
P04350	tubulin beta 4A class IVa	Homo Sapiens	TUBB4A_HUMAN
P48169	gamma-aminobutyric acid type A receptor subunit alpha4	Homo Sapiens	GABRA4_HUMAN
P18507	gamma-aminobutyric acid type A receptor subunit gamma2	Homo Sapiens	GABRG2_HUMAN
P04150	nuclear receptor subfamily 3 group C member 1	Homo Sapiens	NR3C1_HUMAN
Q96SW2	cereblon	Homo Sapiens	CRBN_HUMAN
P14778	interleukin 1 receptor type 1	Homo Sapiens	IL1R1_HUMAN
P01008	serpin family C member 1	Homo Sapiens	SERPINC1_HUMA

			N
P22894	matrix metallopeptidase 8	Homo Sapiens	MMP8_HUMAN
Q13885	tubulin beta 2A class IIa	Homo Sapiens	TUBB2A_HUMAN
Q9BVA1	tubulin beta 2B class IIb	Homo Sapiens	TUBB2B_HUMAN
P09237	matrix metallopeptidase 7	Homo Sapiens	MMP7_HUMAN
P45452	matrix metallopeptidase 13	Homo Sapiens	MMP13_HUMAN
Q13619	cullin 4A	Homo Sapiens	CUL4A_HUMAN
P17181	interferon alpha and beta receptor subunit 1	Homo Sapiens	IFNAR1_HUMAN
P30556	angiotensin II receptor type 1	Homo Sapiens	AGTR1_HUMAN
Q16445	gamma-aminobutyric acid type A receptor subunit alpha6	Homo Sapiens	GABRA6_HUMAN
P47870	gamma-aminobutyric acid type A receptor subunit beta2	Homo Sapiens	GABRB2_HUMAN
P23415	glycine receptor alpha 1	Homo Sapiens	GLRA1_HUMAN
P08913	adrenoceptor alpha 2A	Homo Sapiens	ADRA2A_HUMAN
P47869	gamma-aminobutyric acid type A receptor subunit alpha2	Homo Sapiens	GABRA2_HUMAN
P48551	interferon alpha and beta receptor subunit 2	Homo Sapiens	IFNAR2_HUMAN
P08887	interleukin 6 receptor	Homo Sapiens	IL6R_HUMAN
Q8N1C3	gamma-aminobutyric acid type A receptor subunit gamma1	Homo Sapiens	GABRG1_HUMAN
P18505	gamma-aminobutyric acid type A receptor subunit beta1	Homo Sapiens	GABRB1_HUMAN
P28472	gamma-aminobutyric acid type A receptor subunit beta3	Homo Sapiens	GABRB3_HUMAN
Q16531	damage specific DNA binding protein 1	Homo Sapiens	DDB1_HUMAN

Q9NPC2	potassium two pore domain channel subfamily K member 9	Homo Sapiens	KCNK9_HUMAN
P06213	insulin receptor	Homo Sapiens	INSR_HUMAN
O14649	potassium two pore domain channel subfamily K member 3	Homo Sapiens	KCNK3_HUMAN
Q9BUF5	tubulin beta 6 class V	Homo Sapiens	TUBB6_HUMAN
Q99928	gamma-aminobutyric acid type A receptor subunit gamma3	Homo Sapiens	GABRG3_HUMAN
P18825	adrenoceptor alpha 2C	Homo Sapiens	ADRA2C_HUMAN
P31644	gamma-aminobutyric acid type A receptor subunit alpha5	Homo Sapiens	GABRA5_HUMAN
Q7Z418	potassium two pore domain channel subfamily K member 18	Homo Sapiens	KCNK18_HUMAN
O14764	gamma-aminobutyric acid type A receptor subunit delta	Homo Sapiens	GABRD_HUMAN
P68371	tubulin beta 4B class IVb	Homo Sapiens	TUBB4B_HUMAN
P07437	tubulin beta class I	Homo Sapiens	TUBB_HUMAN
P03956	matrix metallopeptidase 1	Homo Sapiens	MMP1_HUMAN
Q9NYK1	toll like receptor 7	Homo Sapiens	TLR7_HUMAN
P27487	dipeptidyl peptidase 4	Homo Sapiens	DPP4_HUMAN
P15509	colony stimulating factor 2 receptor subunit alpha	Homo Sapiens	CSF2RA_HUMAN
Q9NR96	toll like receptor 9	Homo Sapiens	TLR9_HUMAN
Q13509	tubulin beta 3 class III	Homo Sapiens	TUBB3_HUMAN
Q3ZCM7	tubulin beta 8 class VIII	Homo Sapiens	TUBB8_HUMAN
P18089	adrenoceptor alpha 2B	Homo Sapiens	ADRA2B_HUMAN

Supplementary Table S3: Number of unique ligands gathered from PDB, ChEMBL, PubChem, and IUPHAR for COVID-19 targets from UniProt pre-release web page.

Target shortcut	PDB	ChEMBL	IUPHAR	PubChem	# Unique active compounds
PPIA_HUMAN	57	2	1	3123	3183
CATL1_HUMAN	25	38	4	946	1003
ITAL_HUMAN	13	94	2	550	564
FURIN_HUMAN	4	10	1	448	463
R1AB_CVHSA	37	187	0	47	227
ACE2_HUMAN	4	65	3	161	172
R1A_CVHSA	35	92	0	79	141
SMAD3_HUMAN	3	64	0	65	71
IL6_HUMAN	3	0	0	13	16
DDX1_HUMAN	0	7	0	7	14
R1AB_SARS2	14	0	0	0	9
TMPS2_HUMAN	2	3	4	3	7
IL17_HUMAN	7	0	0	0	7
SPIKE_SARS2	5	0	0	0	5
SPIKE_CVHSA	5	0	0	0	5
BST2_HUMAN	5	0	0	0	5
IL6RB_HUMAN	4	0	0	0	4

BASI_HUMAN	3	0	0	0	3
IMA1_HUMAN	3	0	0	0	3
IL17F_HUMAN	3	0	0	0	3
SGTA_HUMAN	2	0	0	0	2
R1A_SARS2	2	0	0	0	2
VME1_CVHSA	2	0	0	0	2
IL6RA_HUMAN	2	0	0	0	2
I17RC_HUMAN	1	0	1	0	2
I17RA_HUMAN	2	0	0	0	2
MPP5_HUMAN	1	0	0	0	1
ORF9B_CVHSA	1	0	0	0	1
I17RC_HUMAN	1	0	0	0	1
FYV1_HUMAN	0	0	1	0	1

Supplementary Table S4: Number of unique ligands gathered from PDB, ChEMBL, PubChem, and IUPHAR for COVID-19 targets from the Open Targets Platform.

Target shortcut	ChEMBL	IUPHAR	PubChem	PDB	# Unique active compounds
GABRG2_HUMAN	152	0	677	2	831
MMP13_HUMAN	319	3	80	28	430
GABRB1_HUMAN	121	0	166	0	287
DPP4_HUMAN	140	2	29	14	185
GABRA1_HUMAN	3	4	172	2	181
ADRA2C_HUMAN	60	19	99	0	178
AGTR1_HUMAN	111	14	34	1	160
MMP1_HUMAN	57	1	65	9	132
MMP8_HUMAN	81	2	12	18	113
TUBB2A_HUMAN	53	0	56	0	109
GABRG1_HUMAN	49	0	52	0	101
GABRP_HUMAN	46	0	54	0	100
NR3C1_HUMAN	79	8	0	1	88
GABRE_HUMAN	38	0	46	0	84
ADRA2A_HUMAN	47	13	13	0	73
GABRG3_HUMAN	33	0	35	0	68
GABRB2_HUMAN	0	0	65	1	66
GABRD_HUMAN	0	0	48	0	48

TUBB2B_HUMAN	20	0	20	3	43
ADRA2B_HUMAN	11	14	16	0	41
GABRA5_HUMAN	33	4	0	1	38
TUBB_HUMAN	0	0	33	3	36
TUBB6_HUMAN	15	0	15	0	30
PTGS2_HUMAN	23	0	4	2	29
INSR_HUMAN	4	3	10	9	26
TUBB4A_HUMAN	0	0	23	0	23
TUBB3_HUMAN	0	0	18	4	22
GLRA1_HUMAN	4	7	2	1	14
MMP7_HUMAN	4	2	0	8	14
CRBN_HUMAN	1	7	2	1	11
KCNK9_HUMAN	1	0	5	4	10
GABRA2_HUMAN	4	4	0	0	8
TUBB1_HUMAN	0	0	8	0	8
PTGS1_HUMAN	1	5	1	0	7
TUBB4B_HUMAN	0	0	7	0	7
KCNK2_HUMAN	0	2	0	3	5
SERPINC1_HUMAN	0	2	0	3	5
TLR7_HUMAN	3	1	1	0	5
KCNK10_HUMAN	0	1	0	2	3

KCNK3_HUMAN	0	2	0	1	3
DDB1_HUMAN	0	0	0	2	2
GABRB3_HUMAN	0	0	0	2	2
IL1R1_HUMAN	0	0	0	2	2
IL6R_HUMAN	0	0	0	2	2
CSF2RA_HUMAN	0	0	0	1	1
CUL4A_HUMAN	0	0	0	1	1
GABRA4_HUMAN	1	0	0	0	1
IFNAR1_HUMAN	0	0	0	1	1
IFNAR2_HUMAN	0	0	0	1	1
RBX1_HUMAN	0	0	0	1	1

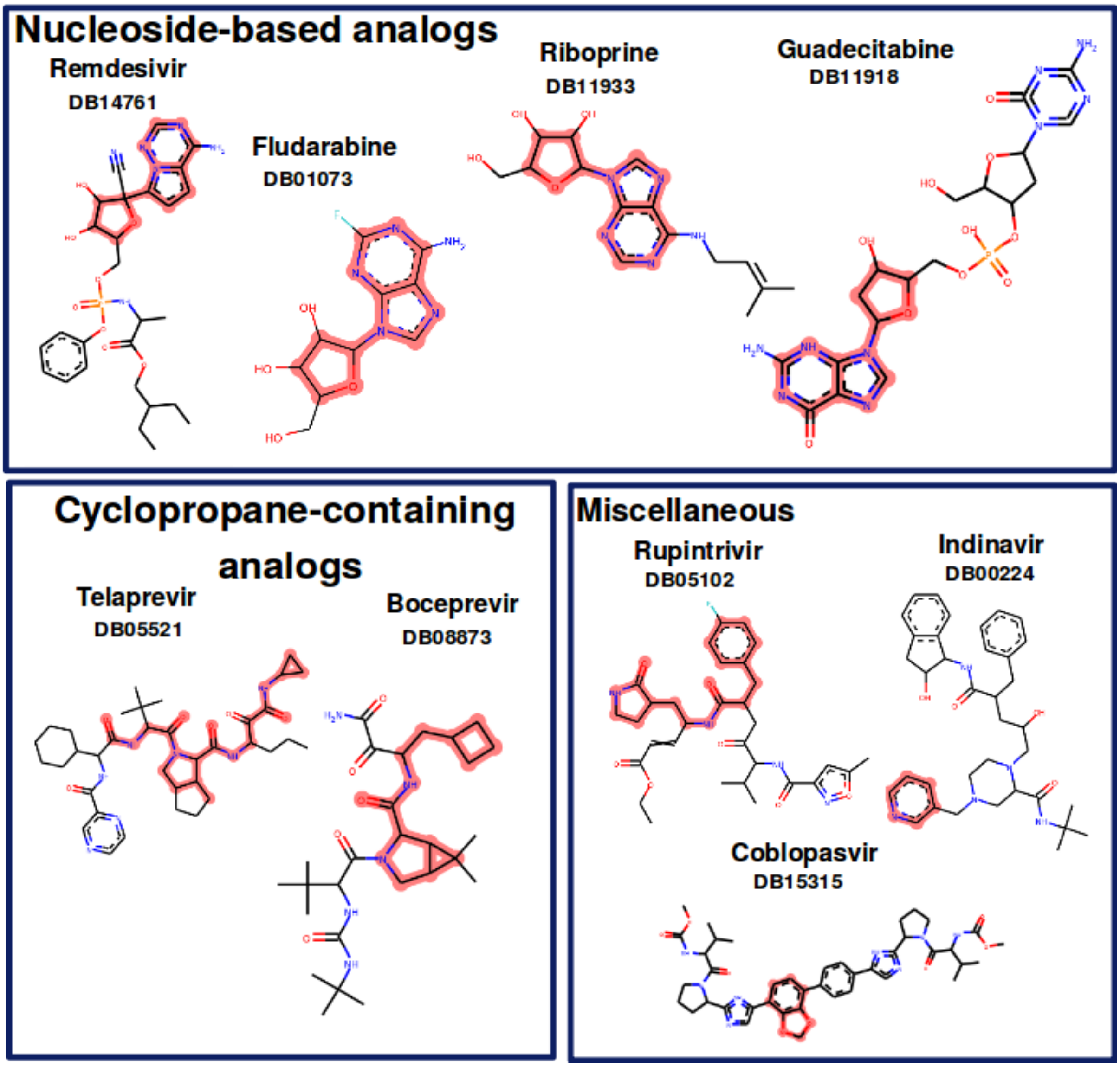

Supplementary Figure 1: *Examples of identified drugs with structural queries highlighted..*

Description of Supplementary Data Files:

Supplementary File S1: CSV file with maximum common substructures in SMARTS format (n= 257) detected via hierarchical scaffold clustering for the COVID-19 use case (available at https://github.com/AlzbetaTuerkova/Drug-Repurposing-in-KNIME/blob/master/Supplementary_file1.csv)

Supplementary File S2: CSV file with identified hits for COVID-19 from DrugBank (n=7,836; available at https://github.com/AlzbetaTuerkova/Drug-Repurposing-in-KNIME/blob/master/Supplementary_file2.csv)

Supplementary File S3: CSV file with identified hits for COVID-19 from CAS Dataset (n=36,521; available at https://github.com/AlzbetaTuerkova/Drug-Repurposing-in-KNIME/blob/master/Supplementary_file3.csv)

Supplementary File S4: CSV file with identified for COVID-19 hits by both DrugBank and CAS Dataset (n=228; available at https://github.com/AlzbetaTuerkova/Drug-Repurposing-in-KNIME/blob/master/Supplementary_file4.csv)

Supplementary File S5: CSV file with identified hits for GLUT-1 deficiency syndrome from DrugBank (n=539; available at https://github.com/AlzbetaTuerkova/Drug-Repurposing-in-KNIME/blob/master/Supplementary_file5.csv)

Supplementary File S6: KNIME drug repurposing workflow (KNWF file) where external UniProt dataset is used as an input(available at https://github.com/AlzbetaTuerkova/Drug-Repurposing-in-KNIME/blob/master/DrugRepurposingPipeline_UniProt.knwf)

Supplementary File S7: KNIME drug repurposing workflow (KNWF file) where disease-target associations from the OpenTarget platform are used an input (available at https://github.com/AlzbetaTuerkova/Drug-Repurposing-in-KNIME/blob/master/DrugRepurposingPipeline_OpenTargets.knwf)

Supplementary File S8: A .pdf Tutorial file "Part 1: Programmatic access to UniProt database using KNIME" (available at https://github.com/AlzbetaTuerkova/Drug-Repurposing-in-KNIME/blob/master/Part1.pdf)

Supplementary File S9: A .pdf Tutorial file "Part 2: Using cross-references to retrieve structural data from the Protein Data Bank (PDB)" (available at https://github.com/AlzbetaTuerkova/Drug-Repurposing-in-KNIME/blob/master/Part2.pdf)

Supplementary File S10: A .pdf Tutorial file "Part 3: Integrative data mining of ligand bioactivity data from ChEMBL and PubChem" (available at https://github.com/AlzbetaTuerkova/Drug-Repurposing-in-KNIME/blob/master/Part3.pdf)

Supplementary File S10: A .pdf Tutorial file "Part 4: Substructure searches in DrugBank" (available at https://github.com/AlzbetaTuerkova/Drug-Repurposing-in-KNIME/blob/master/Part4.pdf)

Supplementary File S11 A .pdf Tutorial file with the answer sheet (available at https://github.com/AlzbetaTuerkova/Drug-Repurposing-in-KNIME/blob/master/Answersheet.pdf)
